# Activation of the Lactate Receptor GPR81 Ameliorates Senescence Hallmarks and Improves Muscle Function in Cellular and Progeroid Models of Aging

**DOI:** 10.1111/acel.70647

**Published:** 2026-08-02

**Authors:** Pihu Mehrotra, Sai Harsha Bhamidipati, Pedro Lei, John Toftegaard, Debanik Choudhury, Maryam Elsayed, Yali Zhang, Jianmin Wang, Shweta Chitkara, G. Ekin Atilla‐Gokcumen, Song Liu, Stelios T. Andreadis

**Affiliations:** ^1^ Department of Chemical and Biological Engineering University at Buffalo Buffalo New York USA; ^2^ Department of Biomedical Engineering University at Buffalo Buffalo New York USA; ^3^ Department of Biostatistics and Bioinformatics Roswell Park Comprehensive Cancer Center Buffalo New York USA; ^4^ Department of Chemistry University at Buffalo Buffalo New York USA; ^5^ Center of Excellence in Bioinformatics and Life Sciences Buffalo New York USA; ^6^ Center for Cell, Gene and Tissue Engineering (CGTE), University at Buffalo Buffalo New York USA

**Keywords:** aging, lipids, metabolism, mitochondria, sarcopenia, skeletal muscle

## Abstract

Skeletal muscle aging is associated with increased lipid accumulation, or myosteatosis, leading to lipotoxicity and loss of muscle function. Here, we report that loss of the lactate receptor GPR81 in cellular and progeroid models of muscle aging is associated with impaired lipid oxidation and enhanced lipid accumulation. Knockdown of GPR81 in young healthy myoblasts led to an increase in senescence hallmarks such as DNA damage, accumulation of reactive oxygen species (ROS), impaired mitochondrial activity, and autophagy. Conversely, treatment of senescent myoblasts with GPR81 agonists enhanced lipid oxidation, leading to a decrease in lipid accumulation, ultimately resulting in decreased DNA damage, ROS accumulation, and enhanced ability to form myotubes. In agreement with our in vitro findings, we observed significant improvement in muscle regeneration and overall health of progeric mice that were treated with GPR81 agonists. Our findings suggest that GPR81 plays a key role in skeletal muscle lipid metabolism, and agonists of GPR81 might play a promising role in reversing age‐associated lipid accumulation and loss of muscle function.

## Graphical abstract

Skeletal muscle aging is associated with a decrease in expression of the lactate receptor GPR81. Treating senescent myoblasts in vitro and progeric mice in vivo with GPR81 agonist decreases intramyocellular lipid accumulation and reverses hallmarks of aging.

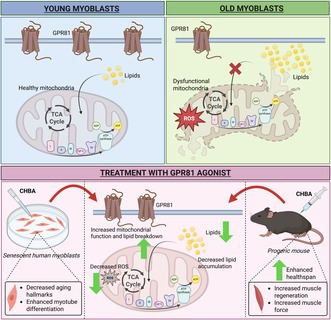

## 1 Introduction

Skeletal muscle aging or sarcopenia results in loss of muscle mass and strength, causing significant muscle weakness in older adults (Walston 2012; Sato et al. 2020). It has been well established that muscle aging is a result of dysregulated metabolism and is associated with mitochondrial dysfunction (Chen et al. 2023; Seo et al. 2016), enhanced accumulation of reactive oxygen species (ROS) (Chen et al. 2022) and impaired glycolysis (Lanza et al. 2007). All these markers of muscle aging are closely linked to alterations in lipid metabolism and increased lipid accumulation in aged muscle (Al Saedi et al. 2022; Starling 2024; Laurentius et al. 2019).

Lipid breakdown occurs in mitochondria to convert fatty acids to acyl‐CoA and generate energy in the form of ATP. As a result, metabolically active organs such as the skeletal muscle rely heavily on lipids for their function (Chung 2021). With aging and other metabolic disorders such as obesity, lipids from the adipose tissues infiltrate the skeletal muscle and deposit as intramyocellular lipid droplets (IMCLs) or inter‐ and intramuscular adipose tissue (IMAT), leading to lipotoxicity and myosteatosis (Kim and Kim 2021). IMCLs and IMATs have been associated with insulin resistance and an increased risk of developing type 2 diabetes (Goodpaster et al. 2000, 2003), loss of muscle strength and mobility (Tuttle et al. 2012; Goodpaster et al. 2001) and poor oncological outcomes (Aleixo et al. 2020). Hence, understanding how lipids affect the cellular and molecular pathways involved in skeletal muscle function, and finding ways to deplete IMCLs and IMATs, is crucial to aid healthy aging and prevent loss of muscle function in older adults.

The adipose tissue is the largest energy reservoir and endocrine organ in the body, and is responsible for controlling lipid storage, mobilization, and distribution (Wang et al. 2022; Luo and Liu 2016). Recent work has established that the lactate receptor, GPR81, plays a crucial role in adipocyte lipid metabolism. Specifically, it has been shown that lactate suppresses lipolysis in the adipose tissue through direct activation of GPR81 (Liu et al. 2009). More recently, Nordström et al. (2023) demonstrated that GPR81 is also expressed in human skeletal muscle, predominantly in type II glycolytic fibers and to a lesser extent in type I oxidative fibers. While the role of GPR81 in skeletal muscle function has not been elucidated, it is well established that the GPR81‐ligand lactate is crucial for skeletal muscle function. In C2C12 myoblasts, supplementation with extracellular lactate resulted in muscle hypertrophy through activation of the ERK1/2 signaling pathway (Ohno et al. 2018). Further, lactate administration resulted in enhanced proliferation and differentiation of myoblasts by activating the AMPK signaling pathway (Zhou et al. 2022). Oral administration of lactate in a mouse model led to an increase in muscle weight, fiber cross section area, and enhanced muscle regeneration by increasing proliferation of Pax7‐positive satellite cells (Ohno et al. 2019). Lactate also plays a key role in governing stem cell metabolism (Mehrotra et al. 2023), regulating gene expression through histone lactylation (Zhang et al. 2019), and promoting mitochondrial biogenesis in skeletal muscle following long‐term exposure (Brooks 2020). However, the role of lactate‐mediated GPR81 signaling in skeletal muscle lipid metabolism remains elusive.

In this study, we investigated the role of GPR81 in cellular and progeroid models of skeletal muscle aging. We demonstrate that GPR81 regulates mitochondrial function and lipid metabolism, and that activating GPR81 may be crucial for enhancing skeletal muscle regeneration in these biological models of aging.

## 2 Materials and Methods

### 2.1 Human Myoblast Cell Culture

Human myoblasts from four donors (14‐year‐old female, 18‐year‐old male, 68‐year‐old male, and 75‐year‐old female) were purchased from Cook Myosite (Pittsburgh, PA). The cells were seeded on Matrigel (0.1 mg/mL, CORNING, Corning, NY) coated T‐175 flasks and expanded in skeletal muscle cell growth medium (SkGM) composed of high‐glucose Dulbecco's Modified Eagle's Medium (DMEM; Gibco, Grand Island, NY) supplemented with 20% (v/v) fetal bovine serum (FBS; Atlanta Biologicals, Flowery Branch. GA), bovine serum albumin (BSA; 0.5 mg/mL; VWR, Radnor, PA), epidermal growth factor (EGF, 10 ng/mL; Lonza, Morristown, NJ), basic fibroblast growth factor (bFGF, 1 ng/mL; Isokine, Iceland), dexamethasone (0.2 μg/mL; VEDCO, Saint Joseph, MO), gentamycin (10 μg/mL; Gibco), 1% Antibiotic‐Antimycotic (Gibco). To generate a model of replicative senescence, the cells were cultured over multiple passages in a humidified incubator at 37°C and 10% CO_2_, and the medium was replenished every other day. Cells were passaged every 4–5 days when they reached 80% confluence. Cells cultured for 1–4 passages (< 8 population doublings) were termed as young myoblasts (Y). Cells cultured for > 10 passages (> 20 population doublings) were termed as senescent myoblasts (S).

Human myoblasts cultured for more than 9 passages (P9) have been previously characterized to exhibit well‐known markers of cellular senescence (Shahini et al. 2021). P9 senescent myoblasts were treated with GPR81 agonists 3‐chloro‐5‐hydroxy BA (CHBA; 100 μM; Cayman Chemical, Ann Arbor, MI) or GPR81 agonist 1 (compound 2/C2; 5 μM; MedChem Express, NJ) for 10 days supplemented in SkGM. Fresh medium was replenished with the agonists every other day. Cells were passaged twice when they reached 80% confluence, and all experiments were performed after 10 days of treatment with the agonists when they were at passage 11 (P11). Untreated P11 myoblasts served as senescent controls (S). Young myoblasts (P2‐4) served as young controls (Y).

### 2.2 shRNA Vectors for *GPR81* Knockdown

MISSION shRNA bacterial glycerol stock that carried either the empty vector (pLKO.1‐Puro, SHC001, Sigma‐Aldrich) or *GPR81*‐specific shRNA (NM_032554, TRCN0000008942, Sigma‐Aldrich) was purchased. Bacteria glycerol stocks were expanded, and plasmids were extracted using the NucleoBond Xtra Midi kit (Cat # 740410, Macherey‐Nagel, Allentown, PA).

To confirm specific knockdown of *GPR81*, shRNA targeting different sequences in *GPR81* was designed using Block‐iT RNA Designer (Thermo Fisher Scientific, Waltham, MA). Oligos containing the shRNA sequences targeting *GPR81* (both sense and anti‐sense), the short hairpin, a termination sequence, as well as nucleotide sequences matching digested EcoRV and ClaI sites were synthesized (Table 1, Thermo Fisher Scientific). A PmeI site was included between the termination sequence and ClaI for confirmation of successful cloning. Complimentary oligos were first annealed (95°C, 3 min; 72°C, 2 min; 50°C, 2 min; 37°C, 2 min; and 25°C, 2 min) and then cloned into the shLVDP vector that was previously developed in our laboratory (Alimperti et al. 2012). The resulting vector was then expanded in STBL3 (Cat # 737303, Thermo Fisher Scientific) and plasmids were isolated as described above.

**TABLE 1:** shRNA sequences targeting GPR81.

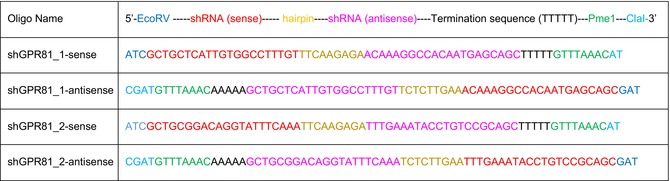

### 2.3 Lentivirus Production

Three plasmids (shRNA encoding lentiviral vector, psPAX2 [plasmid # 12260, Addgene, Watertown, MA] and pMD2.G [plasmid # 12259, Addgene]) were used to co‐transfect 293 T cells using the standard calcium phosphate precipitation method for lentivirus production. The virus was harvested 48 h post‐transfection after using sodium butyrate (5 mM, Sigma‐Aldrich) to increase the lentiviral vector titer. The virus was filtered through a 0.45 μm filter (Corning) and pelleted by centrifugation (50,000× *g* at 4°C for 2 h). Finally, the pellet was resuspended in fresh DMEM and stored at −80°C until use.

### 2.4 Stable Knockdown of GPR81 in Human Myoblasts

Young human myoblasts (passage 1–3) were transduced with lentiviral particles that carried either the empty vector (pLKO.puro or empty shLVDP vector; both referred to as Y_Empty) or shRNA targeting GPR81 (vector purchased from Sigma labeled as Y_shGPR81; shLVDP‐shGPR81_1 referred to as Y_KD1; shLVDP‐shGPR81_2 referred to as Y_KD2) in the presence of 8 μg/mL polybrene (Sigma‐Aldrich). The medium was replaced at 4–6 h after transduction, and the cells were cultured in the presence of 1 μg/mL puromycin for 5 days (Cat # BML‐GR312, Enzo Life Sciences, Farmingdale, NY) to select for puromycin‐resistant clones. Knockdown of GPR81 was verified by RT‐qPCR and western blotting.

### 2.5 RNA Isolation, cDNA Synthesis and Real‐Time PCR

Total RNA from human myoblasts was extracted using RNeasy Plus Mini Kit (Cat # 74134, Qiagen) as per manufacturer specified protocol. High‐Capacity cDNA Reverse Transcription Kit (Cat # 4368814, Applied Biosystems, Waltham, MA) was used to generate cDNA from 1 μg of isolated RNA per sample. To assess gene expression, real‐time PCR was performed using SYBR Select Master Mix (Cat # 4472908, Applied Biosystems) using the primer pairs listed in Table 2. Relative gene expression levels were calculated by the ΔΔ*C*_*t*_ method.

**TABLE 2:** List of primers.

Target gene	Forward 5′→3′	Reverse 5′→3′
ACSL1	CTTATGGGCTTCGGAGCTTTT	CAAGTAGTGCGGATCTTCGTG
ACSL3	GCCGAGTGGATGATAGCTGC	ATGGCTGGACCTCCTAGAGTG
ACSL4	CATCCCTGGAGCAGATACTCT	TCACTTAGGATTTCCCTGGTCC
ACSL6	GCACGGCGATCTGTGATTG	GGCGGAACACCTGGTACAT
FABP1	AAGACAGTGGTTCAGTTGGAAG	TGAGTTCGGTCACAGACTTGAT
FABP5	TGAAGGAGCTAGGAGTGGGAA	TGCACCATCTGTAAAGTTGCAG
FABP6	GCCCGCAACTTCAAGATCG	CCTTGCCAACAGTGAACTTGT
CD36	GGCTGTGACCGGAACTGTG	AGGTCTCCAACTGGCATTAGAA
CPT1A	TCCAGTTGGCTTATCGTGGTG	TCCAGAGTCCGATTGATTTTTGC
CPT1B	GCGCCCCTTGTTGGATGAT	CCACCATGACTTGAGCACCAG
CPT2	CTGGAGCCAGAAGTGTTCCAC	AGGCACAAAGCGTATGAGTCT
ATGL	ATGGTGGCATTTCAGACAACC	CGGACAGATGTCACTCTCGC
HSL	TCAGTGTCTAGGTCAGACTGG	AGGCTTCTGTTGGGTATTGGA
MGL	ATGCCAGAGGAAAGTTCCCC	CGTCTGCATTGACCAGGTG
MT‐TL1	CACCCAAGAACAGGGTTTGT	TGGCC ATGGGTATGTTGTTA
β2M	GAGGCTATCCAGCGTACTCCA	CGGCAGGCATACTCATCTTTT
GPR81	CCTTCAAGATTGTTTGGAGCCT	CTGGGCAGGTAGCATGTGAT
PAX7	ACCCCTGCCTAACCACATC	GCGGCAAAGAATCTTGGAGAC
MYF5	CTGCCAGTTCTCACCTTCTGA	AACTCGTCCCCAAATTCACCC
DES	GAGACCATCGCGGCTAAGAAC	GTGTAGGACTGGATCTGGTGT
MRF4	GGAGCGCCATCAGCTATATTG	ATCCGCACCCTCAAGATTTTC
MEF2C	CCAACTTCGAGATGCCAGTCT	GTCGATGTGTTACACCAGGAG
RPL32	GCCCAAGATCGTCAAAAAGAGA	TCCGCCAGTTACGCTTAATTT

### 2.6 Western Blot

To evaluate protein expression levels in human myoblasts, cells were cultured in vitro and lysed using a mixture of Halt Protease Inhibitor (10×; Cat # 1861280, Thermo Fisher Scientific), 41.67 mM dithiothreitol (Cat # 14265, DTT; Cell Signaling Technology, Dancers, MA) and blue loading buffer (3×; Cell Signaling Technology) in DI water. The lysates were then centrifuged for 10 min at 14,000× *g*, and supernatants were collected in separate tubes. Next, samples were denatured at 95°C for 5 min and loaded at equal volumes onto either 10% or 4%–20% Tris‐Glycine SDS‐PAGE gels (Thermo Fisher Scientific). After electrophoresis (90 min, 120 V), proteins were transferred to a 0.45 μm PVDF membrane (Bio‐Rad Laboratories, Hercules, CA), which was blocked for 1 h using 5% (w/v) nonfat dry milk or 5% (w/v) BSA (for detecting phosphorylated proteins) in Tris‐buffered saline (20 mM Tris, 150 mM NaCl) with 0.1% (v/v) Tween 20 detergent (TBST) buffer. Protein expression was detected by incubating the membrane overnight at 4°C with the antibodies listed in Table 3, followed by incubation with horseradish peroxidase–conjugated anti‐mouse or rabbit IgG secondary antibodies (Cell Signaling Technology) for 1 h at room temperature and then exposed to SuperSignal West Pico PLUS chemiluminescence substrate (Cat # 34578, Thermo Fisher Scientific) for 2 min at room temperature. Protein bands were visualized using the ChemiDoc MP imaging system (Bio‐Rad), and the relative expression level of proteins was determined using the NIH ImageJ software.

**TABLE 3:** List of antibodies.

Antibody	Catalog #	Dilution (ICC: immunocytochemistry; IHC: immunohistochemistry; WB: western blot)	Company
GPR81	NLS2095 PA5‐67873	ICC: 1:200 WB: 1:1000	Novus Biotech Invitrogen
ϒH2AX (Phospho‐Histone H2A.X (Ser139))	9718	ICC: 1:100	Cell Signaling Technology
P21	10355‐1‐AP	ICC: 1:200	Proteintech
Ki67	ab245113	ICC: 1:200	Abcam
MYH1	05‐716	ICC: 1:200	Sigma‐Aldrich
ACTN2	ab68167	ICC: 1:100	Abcam
CD36	18836‐1‐AP	ICC: 1:200	Proteintech
Total OxPhos complexes I‐V for murine samples	45–8099	WB: 1:1000	Invitrogen
Total OxPhos complexes I‐V for human samples	45‐8199	WB: 1:1000	Invitrogen
PLIN2	15294‐1‐AP	ICC: 1:200 WB: 1:1000	Proteintech
LAMP1	15665	ICC: 1:200	Cell Signaling Technology
LC3A/B	12741	WB: 1:1000	Cell Signaling Technology
LAMININ	L9393	ICC: 1:500	Sigma‐Aldrich
eMYHC	Sc‐53091	ICC: 1:100	Santa Cruz Biotechnology
PAX7	AB_528428	ICC: 1:100	Developmental Studies Hybridoma Bank
GAPDH	2118	WB: 1:10000	Cell Signaling Technology

To evaluate protein expression in muscle tissue lysates, 10 mg of gastrocnemius muscle (GA) was homogenized in 300 μL Pierce RIPA buffer (Cat # 89900, Thermo Fisher Scientific) supplemented with 1× Halt Protease Inhibitor by bead disruption in bead lysis tubes (Cat # GREENR5‐RNA, Next Advance, Troy, NY) using the Bullet Blender Gold tissue homogenizer (Next Advance), which was chilled using dry ice. Protein concentration of samples was determined using the BCA protein assay kit (Cat# 23250, Thermo Fisher Scientific). Lysates were then centrifuged, and 1× blue loading dye and 1× DTT reducing agent were added. Protein was denatured by incubation at 95°C for 5 min (except while evaluating OxPhos complex expression, where denaturing temperature was reduced to 50°C as per manufacturer protocol), and proteins were loaded at 15 μg per lane in either 10% or 4%–20% Tris‐Glycine SDS‐PAGE gels. Experimental conditions for electrophoresis and antibody incubation, imaging, and analysis were performed as described above. Uncropped scans of all western blots have been provided as Figures S8A–D and S9A–E for transparency.

### 2.7 Senescence‐Associated β‐Galactosidase Assay (SA‐β‐ Gal)

SA‐β‐Gal activity was detected using the Senescence Detection Kit as per manufacturer's protocol (Cat # ab65351; Abcam, Cambridge, UK). In short, cultured cells were washed once with PBS followed by fixation using 4% (w/v) paraformaldehyde (Sigma‐Aldrich) for 10 min at room temperature. The cells were then stained using SA‐β‐Gal staining solution (filtered using a 22 μm syringe) at 37°C overnight in the absence of supplemental CO_2_. Cells were washed thoroughly with PBS, visualized, and imaged using the Zeiss Axio Observer Z1 inverted microscope with an ORCA‐ER CCD camera (Hamamatsu, Japan). The percentage of SA‐β‐Gal positive cells was determined from multiple fields of view containing approximately 200 cells/sample.

### 2.8 Detection of Mitochondrial Activity Using MitoTracker and TMRM Staining

Live mitochondrial staining was carried out by washing myoblasts once with PBS followed by incubation with 100 nM tetramethylrhodamine methyl ester (TMRM, Cat # T668, Thermo Fisher Scientific), 100 nM MitoTrackerTM Red CMXRos (Cat # M7512, Thermo Fisher Scientific), or 100 nM MitoTrackerTM Green FM (Cat # M46750, Thermo Fisher Scientific) in Live Imaging Solution [Gibco Hanks Balanced Salt solution (without phenol red) supplemented with 10 mM glucose (Cat # 103577–100, Agilent technologies, Santa Clara, CA), and 1 mM pyruvate (Cat # 103578–100, Agilent)] for 30 min at 37°C. Cells were then stained with the Hoechst 33342 nuclear dye at a 1:500 dilution in live imaging solution for 5 min at 37°C. This was followed by washing cells thrice with live imaging solution and visualization of mitochondrial stain using the Zeiss Axio Observer Z1 imaging system. Images were captured, and the mean fluorescence intensity for approximately 200 cells/sample was determined for comparison.

### 2.9 Detection of ROS in Live Cells and Muscle Tissue Samples

To detect ROS in human myoblasts in culture, we used the DCFDA/H2DCFDA‐Cellular ROS Assay Kit (Cat # ab113851, Abcam) as per manufacturer recommended protocol. Live myoblasts were washed once with PBS followed by incubation with 20 μM DCFDA fluorogenic dye in live imaging solution for 30 min at 37°C. Cells were then stained with the Hoechst 33342 nuclear dye at 1:500 dilution in live imaging solution for 5 min at 37°C. This was followed by washing cells thrice with live imaging solution, and imaging using the Zeiss Axio Observer Z1 imaging system. Images were quantified for mean fluorescence intensity for approximately 200 cells/sample.

For detection of ROS in muscle tissue, 10 mg of GA was homogenized in 300 μL mammalian cell lysis buffer (ab179835, Abcam) by bead disruption in bead lysis tubes using the Bullet Blender Gold tissue homogenizer. Protein concentration of samples was determined using BCA protein assay kit. Total free radical presence in 5 μg of each sample was measured using the DCF ROS/RNS Assay Kit (Cat # ab238535, Abcam) as per manufacturer's recommended protocol.

### 2.10 Fatty Acid Uptake Assay

To quantify uptake of palmitate, cultured myoblasts were washed with PBS and incubated with 4 μM fluorescently labeled palmitate dye BODIPY FL C‐16 (Cat # D3821, Thermo Fisher Scientific) in Gibco Hanks Balanced Salt solution (without phenol red) supplemented with 0.167 mM BSA‐Palmitate (Cat # 29558, Cayman Chemical), 2.5 mM Glucose (Agilent) and 0.5 mM Carnitine (Cat # 21489, Cayman Chemical) for 30 min at 37°C. Cells were then stained with the Hoechst 33342 nuclear dye at a 1:500 dilution for 5 min at 37°C. This was followed by washing cells thrice with live imaging solution and imaging using the Zeiss Axio Observer Z1 imaging system. Images were quantified for mean fluorescence intensity in approximately 200 cells/sample.

### 2.11 Visualization of Lysosomes and Autophagy Flux Analysis

To visualize lysosome accumulation in myoblasts, cells were washed once with PBS followed by incubation with 1 μM LysoTracker Red DND‐99 dye (Cat # L7528, Thermo Fisher Scientific) in live imaging solution for 20 min at 37°C. Subsequently, the cells were stained with Hoechst 33342 nuclear dye at 1:500 dilution for 5 min at 37°C followed by washing thrice with PBS and imaging using the Zeiss Axio Observer Z1 imaging system. Images were quantified for mean fluorescence intensity in approximately 200 cells/sample.

To quantify the autophagic vesicles, the cells were stained with CYTO‐ID Green autophagy dye according to the manufacturer's protocol (Cat # ENZ‐51031‐0050, Enzo Life Sciences). In short, cells were cultured in starvation medium: glucose‐free, glutamine‐free, and phenol red‐free DMEM (Cat # A1443001, Thermo Fisher Scientific) in the presence or absence of 25 μM chloroquine (Cat # C6628, Sigma‐Aldrich) for 6 h at 37°C. Subsequently, cells were incubated with Cyto‐ID solution for 30 min at 37°C. This was followed by rinsing cells with PBS and trypsinization. Flow cytometry analysis was performed and 10,000 events per sample were acquired using the BD LSRFortessa X‐20 (BD Biosciences, Franklin Lakes, NJ). CYTO‐ID Green histograms were generated using the Alexa Fluor 488 channel. The results were analyzed using FCS Express 6 (De Novo software, Pasadena, CA). Autophagy flux was assessed by measuring the mean of signal recorded in the presence or absence of 20 μM CQ.

$$\begin{array}{cc} \text{Autophagy flux}\;=\; & \left(\mathrm{LC}3A/B-\mathrm{II}\right)/{\left(\text{GAPDH}\right)}_{\text{starv}+\mathrm{CQ}}\\ & -\left(\mathrm{LC}3A/B-\mathrm{II}\right)/{\left(\text{GAPDH}\right)}_{\text{starv}} \end{array}$$

### 2.12 Visualization of Lipid Droplets Using BODIPY and Quantification by Flow Cytometry

To visualize lipid droplets in cultured myoblasts, cells were seeded on chambered cover glass (Cat # 155411PK, Thermo Fisher Scientific) in SkGM overnight. Next day, cells were washed thrice with PBS to get rid of any serum. Lipid droplets were stained by incubating cells in 5 μM BODIPY dye (Cat # D3922, Thermo Fisher Scientific) in live imaging solution for 30 min at 37°C. Cells were then stained with the Hoechst 33342 nuclear dye at 1:500 dilution in live imaging solution for 5 min at 37°C. This was followed by washing cells thrice with live imaging solution, and visualization of lipid droplets under a confocal microscope (Stellaris 5, Leica Microsystems, Deerfield, IL).

To quantify the extent of lipid accumulation, flow cytometry was performed by acquiring 10,000 events per sample for cells stained with BODIPY using BD LSRFortessa X‐20. Mean intensity of the Alexa Fluor 488 channel was plotted, and analysis was performed using FCS Express 6.

### 2.13 Aggresome Detection Using Proteostat Dye

Aggresome accumulation in myoblasts was detected using the PROTEOSTAT Aggresome detection kit (Cat # ENZ‐51035, Enzo Life Sciences) as per manufacturer recommended protocol. In short, myoblasts were seeded on matrigel‐coated chambered coverglass in SkGM and allowed to adhere overnight. Next day, cells were washed with PBS and fixed with 4% (w/v) paraformaldehyde for 15 min at room temperature. Cells were washed again and permeabilized using 0.1% (v/v) TritonX‐100 (Sigma‐Aldrich) in PBS for 10 min at room temperature. Cells were then washed and stained using the dual detection reagent provided in the kit for 30 min at room temperature. Aggresomes were visualized using the rhodamine detection channel under the Leica Stellaris 5 confocal microscope.

### 2.14 Immunocytochemistry of Fixed Cells

Cells were washed with cold PBS (4°C) once and fixed for 10 min with 4% (w/v) paraformaldehyde, followed by permeabilization with 0.1% (v/v) TritonX‐100 in PBS for 10 min at room temperature. Next, samples were blocked using 5% (v/v) goat serum (Life Technologies) in PBS with 0.01% (v/v) Triton X‐100 for 1 h at room temperature. Immunostaining was performed using primary antibodies listed in Table 3, at 4°C overnight. Subsequently, cells were washed and incubated with secondary antibodies: Alexa 488‐ or Alexa 594‐conjugated anti‐IgG antibody (Life Technologies) for 1 h at room temperature. Nuclei were stained using Hoechst 33342 for 5 min at room temperature (Thermo Fisher Scientific). Cells stained with only secondary antibody served as controls.

### 2.15 Differentiation of Myoblasts to Myotubes

Myoblasts were differentiated to multinucleated myotubes as described previously (Rajabian, Choudhury, et al. 2023). In short, human myoblasts were seeded on Matrigel (0.1 mg/mL) coated dishes at 90% confluency in SkGM. Then, the cells were switched to differentiation medium comprising high‐glucose DMEM supplemented with insulin (10 μg/mL), EGF (10 ng/mL), BSA (500 μg/mL), and gentamicin (50 μg/mL) for a period of 5 days. For CHBA and C2 treated cells, these agonists were removed prior to differentiation. Myotube differentiation efficiency was analyzed by calculating fusion index, determined by the percentage of nuclei inside myotubes over the total number of nuclei.

### 2.16 Measurement of OCR Using Agilent Seahorse XF

Seahorse extracellular flux (XFe96) analyzer (Agilent technologies) was used to measure the oxygen consumption rate (OCR). Human myoblasts were seeded at a density of 5000 cells/well in XFe96 Seahorse culture plates. After 24 h, cells were rinsed with and switched into Seahorse Base Medium (XF DMEM medium, Cat #103575, Agilent technologies) supplemented with 10 mM glucose and 1 mM pyruvate to maintain cell viability. Subsequently, OCR was measured after the addition of 1 μM oligomycin (Cat # O4876, Sigma‐Aldrich), 1.5 μM FCCP (Cat # C2920, Sigma‐Aldrich), and a mixture containing 0.5 μM each of antimycin A (Cat # A8674, Sigma‐Aldrich) and rotenone (Cat # R8875, Sigma‐Aldrich).

To assess OCR through lipid oxidation, cells were seeded at a density of 5000 cells/well inXFe96 Seahorse culture plates. After 24 h, the medium was changed to Substrate‐Limited Buffer which consisted of starvation medium supplemented with 0.5 mM glucose, 1 mM GlutaMAX (Cat # 35050–061, Gibco), 0.5 mM carnitine and 1% FBS for overnight incubation. Next day, medium was changed to supplemented FAO assay buffer 1 h before the start of the assay. Supplemented FAO assay buffer was prepared by dissolving 111 mM sodium chloride (NaCl, Cat # BDH9286, VWR), 4.7 mM potassium chloride (KCl, Cat # BDH9258, VWR), 1.25 mM calcium chloride (CaCl_2_, Cat # BDH9224, VWR), 2 mM magnesium sulphate (MgSO_4_, Cat # 2506–01, VWR), and 1.2 mM sodium dihydrogen phosphate (NaH_2_PO_4_, Cat # 3818–01, VWR) in ultrapure water, further supplemented with 2.5 mM glucose, 0.5 mM Carnitine, and 5 mM HEPES and adjusted pH = 7.4. After 45 min of incubation with the supplemented FAO buffer (and 15 min prior to the start of assay), selected groups were treated with 40 μM etomoxir (Cat # 236020, Sigma‐Aldrich) and the remaining groups with the unsupplemented FAO assay buffer. OCR was measured after the addition of 1 μM oligomycin, 1.5 μM FCCP, and a mixture containing 0.5 μM each of antimycin A and rotenone. All drugs were prepared in unsupplemented FAO assay buffer.

After the Seahorse measurements were completed, cell number was measured using a CyQUANT Cell Proliferation Assay kit (Cat # C7026, Thermo Fisher Scientific), and the OCR values were normalized to cell number. To estimate the contribution of individual mitochondria to cellular respiration, we also report OCR values normalized to both cell number and mitochondrial content in Figure S10.

### 2.17 mtDNA Content Quantification

Total DNA of cultured myoblasts was isolated using the QIAmp DNA Mini Kit (Cat # 51304, Qiagen) according to the manufacturer's instructions. Quantitative real‐time PCR was performed using the SYBR Select Master Mix with 25 ng of DNA used per reaction. mtDNA was quantified using the human primers for mitochondrially encoded tRNALeu (UUR) gene (MT‐TL1), and nDNA was quantified using the human primers for Beta‐2‐Microglobulin (β2M) (Primer pairs listed in Table 2). Both mtDNA and nDNA threshold cycle average values were obtained, and the mtDNA content relative to nDNA was calculated: mtDNA/nDNA = 2^(CTnDNA−CTmtDNA)^.

### 2.18 Oil Red O Staining

Human myoblasts were washed with PBS and fixed using 4% (w/v) paraformaldehyde for 10 min at room temperature. Oil‐Red‐O working solution was prepared by mixing three parts of Oil‐Red‐O stock solution (Cat # O1391, Sigma‐Aldrich) to two parts of DI water, vortexed thoroughly and dissolved at 60°C. The solution was filtered using a 0.22 μm syringe. Cells were incubated in Oil‐Red‐O working solution for 5 min at room temperature, followed by washing five times with PBS. The percentage of Oil‐Red‐O positive cells was determined from multiple fields of view containing approximately 200 cells/sample.

### 2.19 Experimental Animals and Administration of 3‐Chloro‐5‐Hydroxy BA (CHBA)

Lamin‐A Knocked In (LAKI, C57BL/6‐*Lmna* ^G609G/G609G^) mice were kindly donated by Dr. Dudley Lamming at the University of Wisconsin‐Madison School of Medicine (Madison, Wisconsin, USA) (Osorio et al. 2011). Mixed‐sex homozygous LAKI mice were used in the study. Young wildtype (WT) mice of similar age were used as controls. LAKI mice were intraperitoneally injected with CHBA (10 mg/kg dissolved in 5% (v/v) DMSO in corn oil (Cat # S6701, Selleckchem Houston, TX); abbreviated as LAKI‐CHBA: seven males and six females) or vehicle control (5% (v/v) DMSO in corn oil; LAKI: seven females and six males), three times a week for 4 weeks. Young WT mice (WT: two females, three males) were also administered vehicle (5% (v/v) DMSO in corn oil). Injections were started when the mice were 2.5 months or 10 weeks old. After 4 weeks of CHBA or vehicle administration, cardiotoxin (CTX dissolved in sterile saline; 20 μM; from *Naja mossambica* mossambica; Cat # C9759, Sigma‐Aldrich) was delivered by intramuscular injection to the right Tibialis Anterior (TA) muscle of WT, LAKI, and LAKI‐CHBA mice, at five to six sites along the length of the muscle (~10 μL per site; total volume 50 μL). The left TA of the animal was left uninjured. Muscles were allowed to regenerate for another week, while CHBA or vehicle was continued to be administered intraperitoneally. Muscle tissues were harvested 1 week after CTX injury, when the mice were 3.5–4 months old.

The mice were kept in a controlled environment where they experienced a 12 h cycle of light and darkness from 6:00 and from 18:00, respectively. Room temperature was maintained at 22°C, and the mice had unrestricted access to food and water. The humidity levels were maintained between 30% and 70%. All research involving animals followed approved protocols from the Institutional Animal Care and Use Committee (IACUC) of the University at Buffalo (IACUC #‐ CCE01031Y). These protocols adhered to the Animal Welfare Act, Public Health Service Policy on humane care and use of laboratory animals, and other relevant federal statutes and regulations governing animal experimentation.

### 2.20 Murine Tissue Samples

Muscle tissues from biologically aged mice (C57BL/6;19–24 months) and young mice (C57BL/6; 3–4 months) of mixed sex were kindly provided by the NIA Aged Rodent Tissue Bank (https://www.nia.nih.gov/research/dab/aged‐rodent‐tissue‐bank). Protein was isolated from these tissues as described above and expression of GPR81 was assessed using western blot.

### 2.21 Open Field Activity Test

The open field test (Med Associates Inc., Fairfax, VT) was used to evaluate locomotor activity of WT, LAKI, and LAKI‐CHBA mice. The mice were habituated to the laboratory environment one day before the test. To conduct open field activity, mice were gently placed in the center of the activity test chamber, which was a transparent square cage (L × W × H: 27.3 × 27.3 × 20.3 cm). The mice were allowed to freely explore the cage for 30 min, and animal movement was tracked using three 16‐beam IR arrays located on both the *X* and *Y* axes for positional tracking and *Z* axis for rearing detection. The test was conducted when the mice were 2.5 months old to record baseline values and 1 month after CHBA or vehicle administration to record endpoint measurements. Activity of the mice was measured by recording ambulatory distance, ambulatory time, and ambulatory counts during the first 5 min of the open field test.

### 2.22 Citrate Synthase Activity in Muscle Tissue Samples

Citrate synthase (CS) activity was measured using the ab239712 assay kit (Abcam) as per the manufacturer's protocol. Briefly, ~10 mg of GA tissue was homogenized in 100 μL ice‐cold assay buffer by bead disruption in bead lysis tubes using the Bullet Blender Gold tissue homogenizer and centrifuged at 10,000× *g* for 5 min. Samples were loaded at a 1:2 dilution (25 μL lysate + 25 μL assay buffer) alongside a GSH standard curve (0–40 nmol) and a positive control. Absorbance at 412 nm was measured in kinetic mode at 25°C for 40 min using the BioTek Synergy four plate reader. Controls (Lysates + reaction mix without CS substrate) were included for background correction. CS activity was calculated from the linear phase of the reaction (0–20 min), converted to nmol/min using the GSH standard curve, and normalized to the protein concentration as determined by the BCA protein assay kit.

### 2.23 In Vivo Muscle Isometric Force Measurement

Muscle isometric force was measured in live animals as described previously (Rajabian, Ikhapoh, et al. 2023). In short, mice were anesthetized using a mixture of isoflurane (2%) and oxygen (0.6%). Next, the skin above the TA muscle was carefully shaved, and the mice were placed on a heated stage maintained at 37°C by circulating warm water. Ophthalmic lubricant was applied, and the nose was connected to an anesthesia device supplying 2% isoflurane and 0.6% oxygen throughout the experiment. The knee of the mice was clamped using a knee clamp attached to the stage, while the leg was secured onto a footplate connected to a servomotor (1300 A: 3‐in‐1 Whole Animal System—Mouse; Aurora Scientific, ON, Canada). Two needle electrodes were inserted near the knee into the TA muscle. The optimal position for muscle contraction was determined by adjusting the distance between the footplate and knee and stimulating the muscle with a single electrical pulse (25 mA, 0.2 ms pulse width; previously optimized conditions). Once the muscle force ceased to increase, the position was considered the best for muscle contraction. Subsequently, tetanic force was measured by stimulating the muscle with a 500 ms duration and 0.2 ms pulse width at frequencies ranging from 10 to 200 Hz (10, 20, 35, 50, 65, 80, 100, 150, 200 Hz), with a 2 min interval between each stimulation. At the end of the experiments, the mice were returned to their cages with a warming pad and monitored until they regained consciousness and exhibited normal behavior. The data obtained were analyzed using the 611 A Dynamic Muscle Analysis (DMA) software (Aurora Scientific, ON, Canada). Maximum Force was recorded at the stimulation frequency of 150 Hz. Isometric force was recorded before the start of injections to record baseline values, and before and after CTX injury to assess muscle regeneration.

### 2.24 Muscle Tissue Isolation, Embedding and Immunostaining

Mice were euthanized using carbon dioxide (CO_2_) overdose followed by cervical dislocation. The TA, Extensor digitorum longus (EDL), Soleus, GA, Quadricep, and Hamstring muscles were isolated, and connective tissue, if any, was carefully removed. All muscles other than the TA were snap frozen. The TA muscle was gently dried and then immersed in OCT embedding medium (Sakura Finetek, Torrance, CA). Next, the tissues were transferred to a container containing dry ice and 2‐Methylbutane (Sigma‐Aldrich) to freeze the tissues. For staining, 10 μm thickness tissue sections were cut using a cryostat (Leica CM1950, Buffalo Grove, IL) at −20°C and placed on positively charged glass slides (Stellar Scientific). Sections were stored at −80°C.

For immunostaining for eMYHC, Pax7 and Laminin, tissue sections were first washed three times in PBS to remove the OCT embedding medium. Subsequently, they were fixed at room temperature, using chilled methanol for 10 min. The slides were washed thrice with PBS. Next, they were immersed in R‐Buffer A (Electron Microscopy Sciences, Hatfield, PA) for antigen retrieval, with the temperature raised to 121°C for 20 min, followed by gradual cooling. To quench endogenous peroxidase activity, the slides were treated with Tyramide H_2_O_2_ solution (Alexa Fluor 555 Tyramide SuperBoost Kit, Thermo Fisher Scientific; Cat # B40913) for 30 min. Subsequently, samples were blocked with a mixture of 5% (v/v) goat serum and 5% (w/v) BSA in PBS for 1 h, followed by Tyramide Blocking Buffer for another hour, and mouse IgG blocking reagent (MOM, Cat #BMK‐2202, Vectors Lab, Burlingame, CA) for an additional hour, as per the manufacturer's protocol. The tissue sections were then incubated overnight at 4°C with primary antibodies (Table 3) diluted in MOM diluent. The next day, the samples were washed thrice with PBS and stained using goat anti‐mouse IgG secondary antibody in the Tyramide kit as instructed. Next, the samples were stained with Alexa Fluor 488 or 647 conjugated goat anti‐rabbit IgG secondary antibody for 1 h at room temperature. Finally, the nuclei were stained with Hoechst 33342 nuclear dye (1:1000 dilution in PBS) for 5 min at room temperature, followed by three washes in PBS. The slides were coverslipped using ProLong Diamond Antifade Mountant (Cat # P36970, Thermo Fisher Scientific).

### 2.25 Sudan Black B Staining in OCT Embedded Muscle Tissue Sections

Sudan Black B (SBB) staining was performed as per previously published protocol with slight modifications (da Silva et al. 2019). Cryosections were thawed at room temperature for 30 min followed by incubation in phosphate‐buffered saline (PBS) for 10 min to remove OCT. Sections were then fixed in 1% (w/v) paraformaldehyde for 5 min at room temperature and washed thrice with DI water. Tissue sections were incubated in 100% ethylene glycol (Cat # BDH1125, VWR) for 7 min. Following this, two drops of SBB solution (Cat # 199664, Sigma‐Aldrich; 7 mg/mL in ethylene glycol filtered through a 0.2 μm filter) were placed on a clean glass slide and the slide containing cryosections was placed facing down in direct contact with SBB drops. Sections were incubated in SBB for 30 min. The slides were then carefully separated and the excess SBB solution was removed by rinsing thrice in DI water. Next, the slides were incubated in 80% ethylene glycol (in DI water) for 7 min. Slides were again rinsed in DI water and coverslipped using ProLong Diamond Antifade Mountant. Images were captured at 10× and 40× magnification with Zeiss Observer Z1? and intensity of SBB per section was quantified using ImageJ.

### 2.26 RNA Sequencing and Analysis

The global gene expression profiles were characterized by next generation RNA sequencing using Illumina platform as previously described (Mehrotra et al. 2024). In short, total RNA was isolated from 10 mg of GA muscle tissue from young WT, LAKI and LAKI‐CHBA mice (*n* = 7 mice per condition) using RNeasy Fibrous Tissue Mini Kit (Catalog # 74704, Qiagen, Valencia, CA) as per manufacturer's instructions. Sequencing libraries were prepared as per standard Illumina protocols (Illumina Stranded Total RNA Prep with Ribo‐Zero Plus), quality checked and quantified by Kapa Biosystems qPCR. The multiplexed libraries were sequenced in pair‐end (2 × 50 bp) on the NovaSeq 6000 at 300 pM with 1% loading control.

Sequenced reads were processed using nf‐core/rnaseq (v3.14.0) pipeline (Ewels et al. 2020; Patel et al. 2024). Quality of the reads was assessed using FastQC (v0.12.1) (Bioinformatics, B 2018). Sequencing reads were trimmed using Trim Galore (v0.6.7) (Krueger et al. 2021) to eliminate the remains of Illumina adaptors and filter reads that were shorter than 20 bp. The filtered reads were then mapped to the mouse genome (GRCm39, Ensembl) using STAR (v2.7.9a) (Dobin et al. 2013) and quantified using Salmon (v1.10.1) (Patro et al. 2017). The abundance estimates were imported into R using tximport (v1.26.1) (Soneson et al. 2015) package. The resulting gene‐level counts were used for differential gene expression and PCA analysis using the DESeq2 (v1.38.3) (Love et al. 2014) package. Genes were considered as differentially expressed if adjusted *p*‐value was < 0.05. Normalized counts matrix from DESeq2, after removal of low‐count genes, was used as input for the GSEA (v4.3.3) software (Subramanian et al. 2005). Mouse gene identifiers were mapped to their human orthologs before the enrichment analysis. Signal‐to‐noise metric was used to rank genes with 1000 permutations with the gene set permutation type and weighted enrichment statistics. Gene set sizes were 15–500 for MSigDB v2024.1.Hs C5:GO and 10–1000 for MSigDB v2024.1.Hs H, C2:CP, C2:CGP. The top 200 gene sets from each phenotype that pass the FDR < 0.25 threshold were considered for visualization. Network representation and clustering of the GSEA data were performed using EnrichmentMap (v3.5.0) (Merico et al. 2010) and AutoAnnotate (v1.5.1)11 (Kucera et al. 2016) in Cytoscape (v3.10.3) (Shannon et al. 2003). All custom scripts used in this study have been deposited to https://github.com/SaiH99/GPR81_RNA_seq repository.

### 2.27 Image Analysis

NIH ImageJ software was used to quantify mean fluorescence intensity (MFI) per cell for protein expression. Briefly, images were converted to 8‐bit and positively stained cells were manually marked and analyzed for Integrated Density. To quantify the number of positively stained cells or muscle fibers, the cell counter plugin on ImageJ was employed.

### 2.28 Statistical Analysis

Statistical significance was assessed by *t*‐test for comparing two samples. For statistical analysis of more than two samples, one‐way or two‐way ANOVA was performed, followed by Tukey post hoc test or Dunnett's multiple comparisons test or Sidak's multiple comparisons test. All analyses were done using the GraphPad Prism 10 software. Values were considered statistically significant for *p* < 0.05. Statistical significance was denoted as *p < 0.05, **p < 0.01, ***p < 0.001, and ****p < 0.0001. All in vitro data are presented as mean ± standard deviation (SD) from independent experiments. Unless otherwise stated, experiments were performed using at least three independent biological donors to ensure reproducibility. For analyses of MFI/cell, individual cell measurements from three independent biological replicates are displayed (shown in lighter colors) to illustrate the distribution of the data, while the mean value of each biological replicate is indicated by darker symbols. Statistical analyses were performed using the mean values from independent biological replicates. The *p*‐values shown in figures correspond to comparisons between biological replicates (p_b.r_.). Where relevant, *p*‐values calculated from individual cell measurements are additionally reported in the text (p_i.c_.) to illustrate cell‐to‐cell variability. All in vivo data were represented as mean ± SD and graphs were plotted as dispersion plots with each dot representing an animal.

## 3 Results

### 3.1 Impaired Lipid Oxidation and Myosteatosis Are Hallmarks of Senescent Skeletal Muscle

**FIGURE 1:**
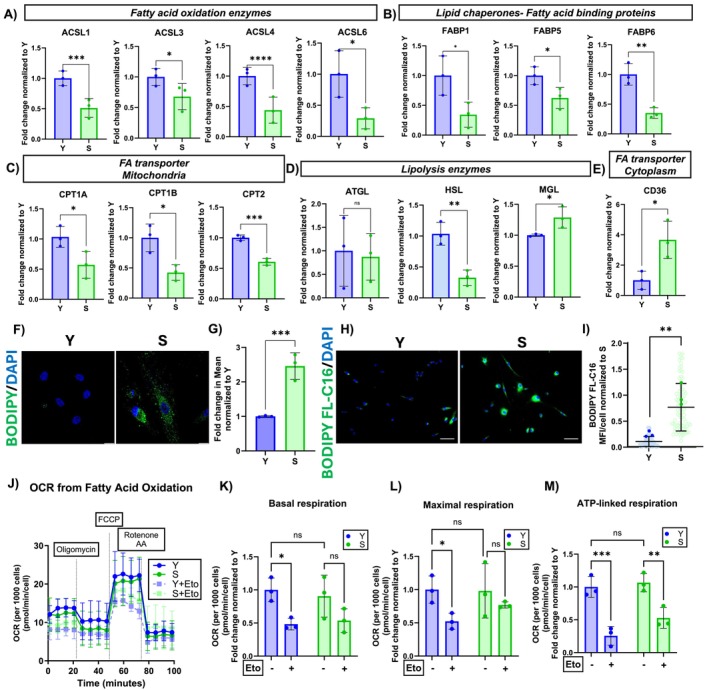
Impaired lipid oxidation and enhanced lipid accumulation are hallmarks of skeletal muscle senescence. Quantitative real‐time PCR for key enzymes governing (A) Fatty acid oxidation; *ACSL1*, *ACSL3*, *ACSL4* and *ACSL6*. (B) Fatty acid binding proteins; *FABP1*, *FABP5* and *FABP6*. (C) Mitochondrial transporters of fatty acyl carnitines from cytoplasm to mitochondria; *CPT1A*, *CPT1B* and *CPT2*. (D) Enzymes responsible for lipolysis; *ATGL*, *HSL* and *MGL*. (E) The cytoplasmic fatty acid transport protein *CD36*. Data internally normalized to *RPL32* cycle number followed by normalized to expression in Y. (F) Representative confocal images for BODIPY (green) staining to depict lipid droplets in Y and S myoblasts. Nuclei stained using Hoechst 33342 (blue). Scale bar represents 10 μm. (G) Fold change in mean of BODIPY signal normalized to Y. (H) Representative images of BODIPY FL‐C16 (green) live staining in Y and S cells. Nuclei stained using Hoechst 33342 (blue). Scale bar represents 100 μm. (I) Quantification of BODIPY FL‐C16 intensity per cell, normalized to S; data shown for > 100 cells from three independent biological replicates. (J) Measurements of oxygen consumption rate (OCR) using Seahorse extracellular flux analyzer in the presence or absence of Etomoxir (Eto) in Y and S cells. (K) Basal Respiration, (L) Maximal Respiration and (M) ATP‐linked respiration calculated from OCR data in (J). All data shown as mean ± SD. All experiments were performed using three independent biological replicates. *p < 0.05, **p < 0.01, ***p < 0.001, ****p < 0.0001.

We assessed how lipid metabolism is affected by cellular senescence using a model of replicative senescence of human myoblasts. To this end, we measured the expression levels of key genes involved in lipid transport and metabolism in young (Y) and senescent (S) myoblasts in culture. The long‐chain acyl‐CoA synthetase (ACSL) family of genes—*ACSL1*, *ACSL3*, *ACSL4*, and *ACSL6*—which are essential for fatty acid breakdown (Figure 1A) and the fatty acid‐binding proteins (*FABP1*, *FABP5*, and *FABP6*), which regulate lipid trafficking in the cytoplasm, were downregulated in senescent myoblasts (Figure 1B). Additionally, the CPT enzymes (*CPT1A*, *CPT1B*, and *CPT2*), which catalyze the transport of long‐ and medium‐chain fatty acids from the cytoplasm to the mitochondria (Figure 1C), and one of the lipolysis enzymes, *HSL* (Figure 1D), were also downregulated. On the other hand, *CD36*, a key cytoplasmic fatty acid uptake protein, increased in senescent myoblasts, in agreement with literature (Moiseeva et al. 2023), indicating increased lipid uptake in senescent cells (Figure 1E). These results indicate that enzymes involved in lipid breakdown were downregulated, while genes associated with lipid uptake were elevated in senescent myoblasts.

Next, we examined the presence of intracellular lipids in senescent cells using the lipid droplet dye BODIPY. Confocal images revealed that lipid droplets accumulated throughout the cytoplasm of senescent cells, but not in young cells (Figure 1F). This observation was further confirmed by flow cytometry analysis for myoblasts incubated with BODIPY dye (Figure S1A). Indeed, senescent myoblasts accumulated 2.41 ± 0.37‐fold more BODIPY‐stained lipids as compared to young myoblasts (Figure 1G). To examine whether lipid accumulation was the result of increased fatty acid uptake, we incubated young and senescent myoblasts with palmitate conjugated with the green, fluorescent dye BODIPY‐FL C16, and quantified palmitate uptake by measuring the green fluorescence intensity. Despite the presence of lipid droplets, senescent myoblasts showed increased lipid uptake (Figure 1H,I), prompting us to examine whether they are capable of oxidizing lipids for energy production in the mitochondria.

To this end, we employed the Seahorse XF analyzer to measure the Oxygen Consumption Rate (OCR) in the presence of the CPT‐1 inhibitor etomoxir (Figure 1J). We found that both young and senescent myoblasts exhibited equal levels of basal (Figure 1K), maximal (Figure 1L) and ATP‐linked respiration (Figure 1M). However, these were inhibited by etomoxir to a greater extent in young myoblasts compared to senescent myoblasts, indicating a higher contribution of lipids to OCR in young cells. We also found that senescent myoblasts exhibited increased mitochondrial content as compared to young myoblasts, as determined by qRT‐PCR for mtDNA/nDNA ratio (Figure S1B); and greater expression of the OxPhos Complex III‐ UQCRC2, even though the expression of Complexes I, II, IV, and V remained unchanged when compared to young myoblasts (Figure S1C–H), indicating that the loss of mitochondrial respiration is not contributed by a lack of mitochondria in senescent cells.

These results suggest that young myoblasts actively utilize lipids for energy production and do not accumulate lipid droplets. In contrast, senescent myoblasts uptake greater amounts of lipids but do not use them for mitochondrial oxidation, leading to myosteatosis. Lipid metabolism and mobilization is a characteristic of the adipose tissue, which coordinates closely with the skeletal muscle to regulate lipid homeostasis (Goodpaster and Wolf 2004; Jia and Shan 2022). The lactate receptor GPR81 has been implicated to play a key role in lipolysis in the adipose tissue (Liu et al. 2009; Cai et al. 2008). GPR81 has also been shown to be expressed in the skeletal muscle (Nordström et al. 2023), prompting us to hypothesize that GPR81 may be involved in age‐associated disruption of lipid homeostasis in skeletal muscle.

### 3.2 GPR81 Expression in Muscle Decreases With Aging

**FIGURE 2:**
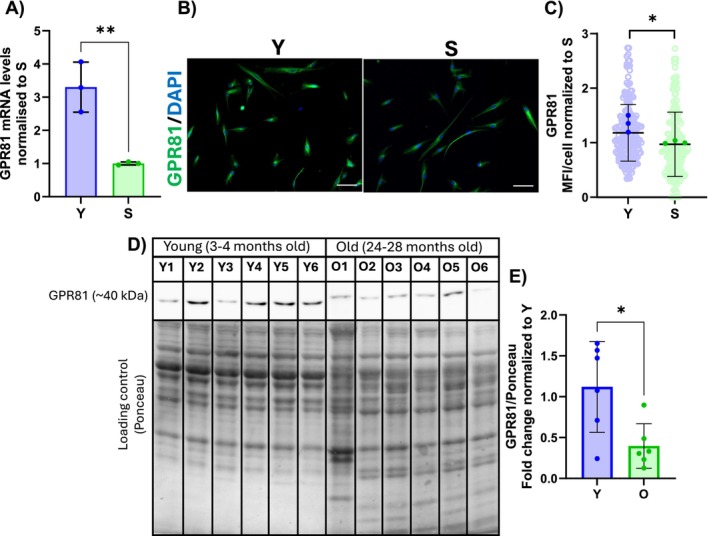
GPR81 expression decreases with aging. (A) Quantitative real‐time PCR for *GPR81* internally normalized to *RPL32* cycle number followed by normalization to S; data shown as mean ± SD. (B) Immunostaining for GPR81 (red) in Y and S myoblasts. Nuclei stained using Hoechst 33342 (blue). Scale bar represents 100 μm. Experiments were performed using three independent biological replicates. (C) Quantification of GPR81 intensity per cell, normalized to S; data shown for > 200 cells from three independent biological replicates. (D) Western blot for GPR81 in muscle isolated from young (3–4 months) and old (24–28 months) mice from *n* = 6 biological replicates, and (E) quantification after normalization to total protein (Ponceau). All data shown as mean ± SD. *p < 0.05, **p < 0.01.

We first assessed if GPR81 expression levels vary as human myoblasts senesce. Indeed, young myoblasts expressed around 4‐fold higher *GPR81* mRNA levels as compared to senescent myoblasts (Figure 2A). Immunostaining for GPR81 showed that GPR81 protein levels were also significantly decreased in senescent myoblasts (1.42 ± 0.57‐fold; Figure 2B,C). To assess whether GPR81 protein expression is affected by organismal aging, we isolated protein from muscle tissues of young (Y, 3–4 months) and old (O, 24–28 months) mice. Western blot analysis revealed higher expression of GPR81 in muscles of young as compared to muscles of old mice (1.68 ± 0.57‐fold; *n* = 6 biological replicates; Figure 2D,E), indicating that GPR81 expression levels decrease as the skeletal muscle ages. We note that the GPR81 band in old samples consistently migrated at a slightly higher apparent molecular weight than that observed in young samples. This shift may reflect differences in posttranslational modification or protein processing; however, the underlying mechanism cannot be determined from the current data.

### 3.3 Loss of GPR81 Enhances Hallmarks of Cellular Senescence

To assess if loss of GPR81 is associated with skeletal muscle senescence, we transduced young myoblasts with lentiviral vectors encoding for shRNAs for GPR81 knockdown (KD). For our experiments, we used two different sets of vectors to knock down GPR81. The first vector, purchased from Sigma‐Aldrich (Y_shGPR81), resulted in ~85% knockdown of GPR81 protein levels (Figure S2A,B) and ~50% decrease in mRNA levels (Figure S2C) in young myoblasts. Myoblasts transduced with the empty vector with the same vector backbone (Y_Empty) served as controls. The second set of vectors consisted of shRNA sequences specific to GPR81 CDS that we inserted into our in‐house designed lentiviral vector (shLVDP) (Alimperti et al. 2012). These were annotated as Y_KD1 and Y_KD2 and resulted in ~50% and ~40% decrease in GPR81 protein levels respectively (Figure S2D,E). Young myoblasts transduced with the same shLVDP vector but with no shRNA served as controls (Y_Empty).

**FIGURE 3:**
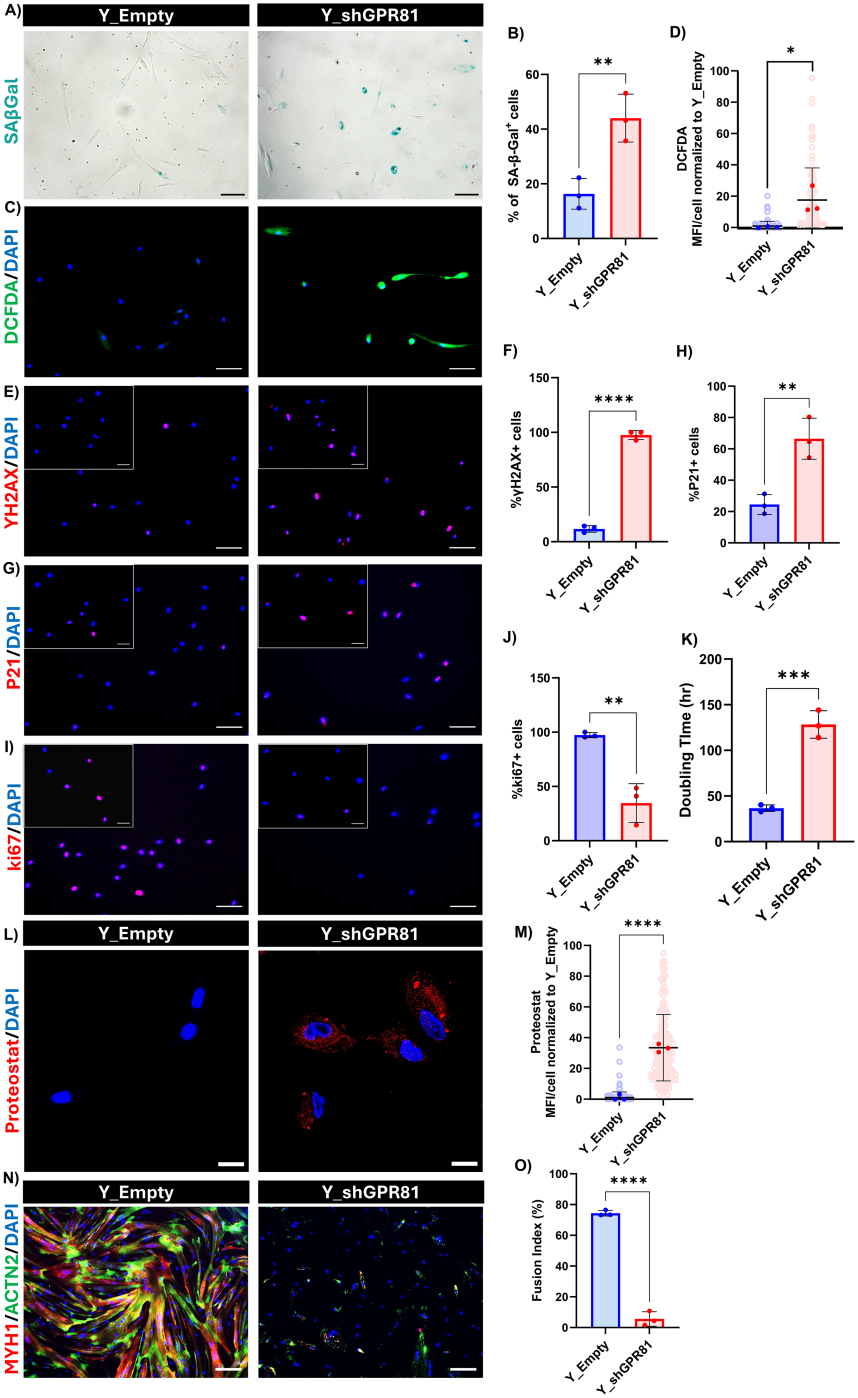
Knockdown of GPR81 in young myoblasts leads to a senescent phenotype. (A) Senescence‐associated β‐galactosidase (SA‐β‐Gal) staining in Y_Empty and Y_shGPR81 cells. Scale bar represents 100 μm. (B) Quantification for percentage of SA‐β‐Gal cells. (C) Representative images of DCFDA (green) live staining. Nuclei stained using Hoechst 33342 (blue). Scale bar represents 100 μm. (D) Quantification of DCFDA intensity per cell, normalized to S; data shown for > 100 cells. (E) Immunostaining for phosphorylated form of histone H2AX (ϒ‐H2AX) (red) in Y_Empty and Y_shGPR81 myoblasts. Nuclei stained using Hoechst 33342 (blue). Scale bar represents 100 μm; Insets at higher magnification with scale bar 50 μm. (F) Quantification of percentage of ϒ‐H2AX positive cells; data shown for > 100 cells. (G) Immunostaining for P21 (red) in Y_Empty and Y_shGPR81 myoblasts. Nuclei stained using Hoechst 33342 (blue). Scale bar represents 100 μm; Insets at higher magnification with scale bar = 50 μm. (H) Quantification of percentage of P21 positive cells, normalized to Y_Empty. (I) Immunostaining for Ki67 (red) in Y_Empty and Y_shGPR81 myoblasts. Nuclei stained using Hoechst 33342 (blue). Scale bar represents 100 μm; Insets at higher magnification with scale bar = 50 μm. (J) Quantification of percentage of Ki67 positive cells, normalized to Y_Empty. (K) Quantification of myoblast doubling time. (L) Representative confocal images for Proteostat (red) staining to depict aggresome accumulation in Y_Empty and Y_shGPR81 myoblasts. Nuclei stained using Hoechst 33342 (blue). Scale bar represents 10 μm. (M) Quantification of proteostat intensity per cell, normalized to Y_Empty; data shown for > 150 cells from three independent biological replicates. (N) Representative images of myoblasts differentiated to myotubes and stained for MYH1 (red) and ACTN2 (green). Nuclei stained using Hoechst 33342 (blue). Scale bar represents 200 μm. (O) Quantification for fusion index after myotube differentiation of Y_Empty and Y_shGPR81 myoblasts. All data shown as mean ± SD. All experiments were performed using three independent biological replicates. *p < 0.05, **p < 0.01, ***p < 0.001, ****p < 0.0001.

GPR81 KD in young myoblasts resulted in increased levels of senescence markers such as senescence associated β‐Galactosidase (SAβGal), a well‐known marker of cellular senescence (Figure 3A,B; Figure S2F,G) and accumulation of ROS (Figure 3C,D; Figure S2H,I). GPR81 KD also compromised genomic integrity as seen by increased levels of phosphorylated histone H2A.X (γH2AX; Figure 3E,F; Figure S2J,K), promoted cell cycle arrest as evidenced by increased levels of nuclear P21 (Figure 3G,H; Figure S2L,M), decreased proliferation as seen by decreased Ki67 staining (Figure 3I,J) and increased doubling times (3.27 ± 0.43‐fold; Figure 3K).

Cellular senescence has been associated with dysregulated proteostasis leading to accumulation of misfolded proteins or aggresomes. Using the proteostat dye (Lima et al. 2023), we assessed changes in protein homeostasis in GPR81 KD cells. Indeed, confocal imaging revealed accumulation of aggresome in the cell cytoplasm of young cells transduced with shGPR81 lentivirus, but not in cells transduced with the empty vector (Figure 3L,M; Figure S2N,O). Since cellular senescence is accompanied by loss of myoblast differentiation potential, we assessed whether GPR81 KD impaired myotube formation. Indeed, while young myoblasts transduced with the empty vector differentiated into myotubes expressing myosin heavy chain 1 (MYH1) and sarcomeric α‐Actinin (ACTN2), GPR81 KD significantly impaired myotube differentiation, as evidenced by decreased expression of MYH1 and ACTN2 (Figure 3N, Figure S2P) and decreased fusion index (Y_Empty: 75.59% ± 2.39%, Y_shGPR81: 6.39% ± 3.78%, Figure 3O; Y_Empty: 61.13% ± 0.76%, Y_KD1: 45.38% ± 2.56%, Y_KD2: 42.91% ± 3.49%, Figure S2Q). To determine whether GPR81 knockdown affects the myogenic identity of proliferating myoblasts, we assessed expression of several myogenic markers by RT‐PCR. We did not detect significant changes in the expression of *PAX7* or *MYF5*, markers associated with proliferating myoblasts. Interestingly, GPR81 knockdown increased expression of *DES*, a marker associated with early myogenic differentiation, while expression of *MEF2C* and *MRF4* remained unchanged (Figure S2R). These results suggest that GPR81 knockdown does not disrupt myoblast identity.

### 3.4 Loss of GPR81 Impairs Mitochondrial Membrane Potential and Respiration

**FIGURE 4:**
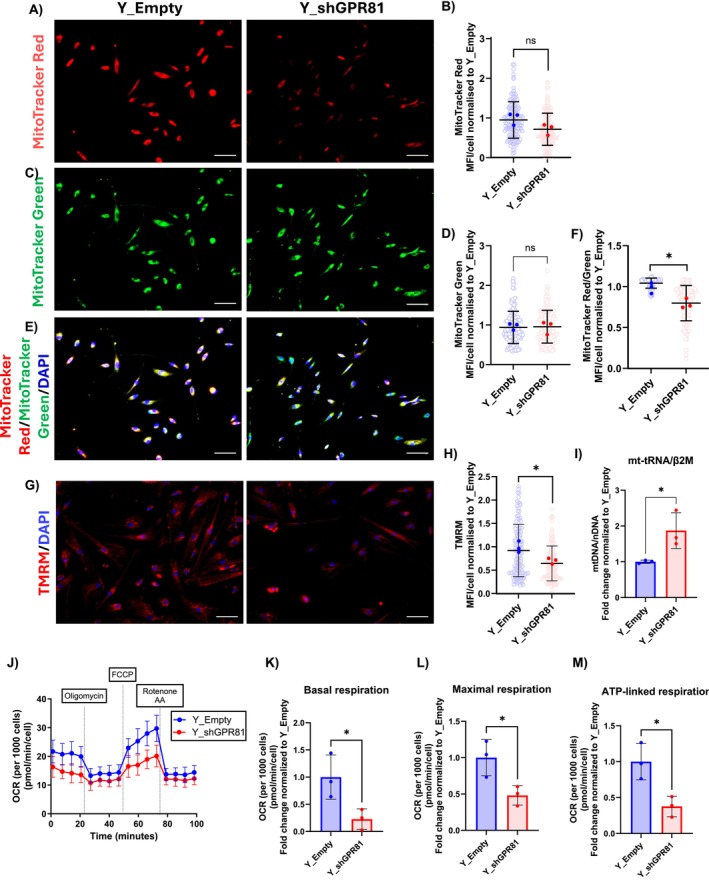
Knockdown of GPR81 in young myoblasts impairs mitochondrial membrane potential. (A, B) Representative images and quantification of MitoTracker Red (red), (C, D) MitoTracker Green and (E) merged images of MitoTracker Red and Green live staining in Y_Empty and Y_shGPR81 cells. Quantifications depict intensity per cell normalized to Y_Empty; data shown for > 100 cells from three independent biological replicates. (F) Quantification for ratio of MitoTracker Red to Green signal for each cell, normalized to Y_Empty; data shown for > 100 cells from three independent biological replicates. (G) Representative images of TMRM (red) live staining in Y_Empty and Y_shGPR81 cells. (H) Quantification of TMRM intensity per cell, normalized to Y_Empty; data shown for > 100 cells from three independent biological replicates. Nuclei for all images stained using Hoechst 33342 (blue). Scale bar (for A, C, E and G) = 100 μm. (I) Quantitative real‐time PCR to assess the level of mtDNA relative to nDNA (mtDNA/nDNA) using primers specific to mitochondrial gene *MT‐TL1* and nuclear gene *β2M*. (J) Measurements of oxygen consumption rate (OCR) using Seahorse extracellular flux analyzer in Y_Empty and Y_shGPR81 cells. (K) Basal Respiration, (L) Maximal Respiration and (M) ATP‐linked respiration calculated from OCR data in (J). All data shown as mean ± SD. All experiments were performed using three independent biological replicates. *p < 0.05.

Mitochondrial dysfunction is a hallmark of skeletal muscle senescence (Shahini et al. 2021). To assess if knockdown of GPR81 in young myoblasts altered mitochondrial function, we co‐stained myoblasts with MitoTracker Red dye, which accumulates in polarized (active) mitochondria and MitoTracker Green, a dye that stains for total cellular mitochondria. GPR81 KD myoblasts exhibited trends of decreased MitoTracker Red intensity (Figure 4A,B; Figure S3A,B; *p*_s.c._: < 0.0001; *p*_b.r._: ns for Figure 4B). While we did not find any significant differences in total mitochondrial content using MitoTracker Green using one set of vectors (Sigma‐Aldrich vector; Figure 4C,D), myoblasts transduced with the shLVDP vectors have slightly altered total mitochondrial content (Figure S3C,D). Nevertheless, the ratio of active to total mitochondria per cell (ratio of Mitotracker Red over Green intensity per cell) was significantly decreased in GPR81 KD cells using two of the vectors (Figure 4E,F; Figure S3E,F; Y_Empty vs. Y_KD1: *p*_s.c._ < 0.0001, *p*_b.r._ = ns). Mitochondrial membrane potential was also assessed using TMRM dye, which also stains active polarized mitochondria in live cells. Knockdown of GPR81 in young myoblasts led to decreased TMRM intensity indicative of decreased mitochondrial membrane potential (Figure 4G,H). Finally, we also observed increased mtDNA/nDNA content in GPR81 KD cells using two of the vectors, indicating increased total mitochondria in these cells (Figure 4I; Figure S3G).

These results prompted us to assess mitochondrial respiration in live cells by measuring Oxygen Consumption Rate (OCR) using the Seahorse XF analyzer (Figure 4J; Figure S3H). We found that basal respiration, maximal respiration and ATP‐liked respiration were downregulated in GPR81 KD myoblasts (Figure 4J–M, Figure S3H–K). Collectively, these data suggest that knockdown of GPR81 in young myoblasts impairs mitochondrial membrane potential and mitochondrial respiration.

### 3.5 Knockdown of GPR81 in Young Myoblasts Impaired Lipid Oxidation

**FIGURE 5:**
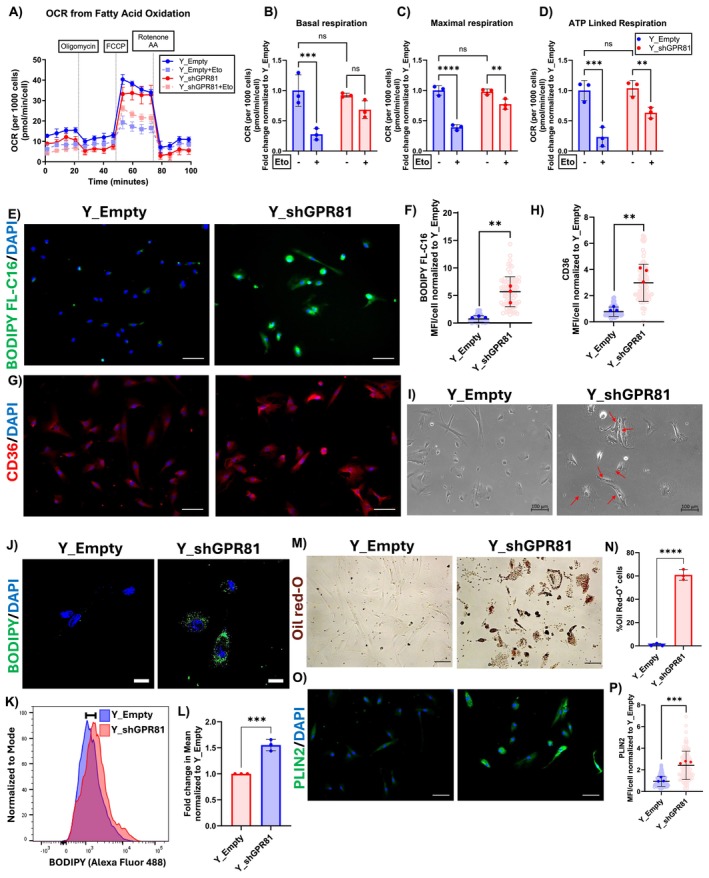
GPR81 knockdown in young myoblasts impairs lipid oxidation and leads to lipid accumulation. (A) Measurements of oxygen consumption rate (OCR) using Seahorse extracellular flux analyzer in the presence or absence of Etomoxir (Eto) in Y_Empty and Y_shGPR81 cells. (B) Basal Respiration, (C) Maximal Respiration and (D) ATP‐linked respiration calculated from OCR data in (A). (E) Representative images of BODIPY FL‐C16 (green) live staining in Y_Empty and Y_shGPR81 cells. Nuclei stained using Hoechst 33342 (blue). Scale bar represents 100 μm. (F) Quantification of BODIPY FL‐C16 intensity per cell, normalized to Y_Empty; data shown for > 100 cells from three independent biological replicates. (G) Immunostaining for CD36 (red) in Y_Empty and Y_shGPR81 myoblasts. Nuclei stained using Hoechst 33342 (blue). Scale bar represents 100 μm. (H) Quantification of CD36 intensity per cell, normalized to Y_Empty; data shown for > 100 cells from three independent biological replicates. (I) Brightfield images depicting lipid droplets in Y_shGPR81 myoblasts (red arrows); scale bar represents 100 μm. (J) Representative confocal images for BODIPY (green) staining to depict lipid droplets in Y_Empty and Y_shGPR81 myoblasts. Nuclei stained using Hoechst 33342 (blue). Scale bar represents 10 μm. (K) Flow cytometry histograms for myoblasts stained with BODIPY (green) and detected using Alexa Fluor 488 channel. (L) Fold change in mean of BODIPY signal normalized to Y_Empty. (M) Representative images of Oil‐Red‐O staining in Y_Empty and Y_shGPR81 myoblasts; scale bar represents 100 μm. (N) Quantification of percentage of Oil‐Red‐O positive cells; data shown for > 100 cells. (O) Immunostaining for PLIN2 (green) in Y_Empty and Y_shGPR81 myoblasts. Nuclei stained using Hoechst 33342 (blue). Scale bar represents 100 μm. (P) Quantification of PLIN2 intensity per cell, normalized to Y_Empty; data shown for > 100 cells from three independent biological replicates. All data shown as mean ± SD. All experiments were performed using three independent biological replicates. *p < 0.05, **p < 0.01, ***p < 0.001, ****p < 0.0001.

Decreased mitochondrial membrane potential and respiration associated with loss of GPR81 prompted us to assess changes in the contribution of fatty acids to OCR in cells with GPR81 knockdown using the Seahorse XF analyzer. Young myoblasts transduced with either empty vector or after GPR81 knockdown has similar levels of basal, maximal and ATP‐linked respiration (Figure 5A–D). However, greater decrease in OCR was observed with etomoxir treatment in cells transduced with empty vector, suggesting that GPR81 knockdown impaired lipid oxidation in myoblasts (Figure 5A–D). Similar to senescent myoblasts, we also observed an increase in uptake of fluorescently labeled palmitate into GPR81 KD cells (Figure 5E,F; Figure S3L,M; Y_Empty vs. Y_KD1: *p*_s.c._ < 0.0001, *p*_b.r._ = ns), which was accompanied by higher expression levels of the fatty acid transporter CD36 (Figure 5G,H; Figure S3N,O; Y_Empty vs. Y_KD1: *p*_s.c._ = 0.0012, *p*_b.r._ = ns). As a result of impaired lipid breakdown and enhanced lipid uptake, we also observed increased lipid accumulation in GPR81 KD myoblasts, as seen in phase contrast imaging (Figure 5I, red arrows) and upon staining with the lipophilic dye, BODIPY (Figure 5J; Figure S3P,Q). To quantify the extent of lipid accumulation, we performed flow cytometry using BODIPY to stain for lipid droplets and found 1.55 ± 0.11‐fold increase in BODIPY fluorescence in GPR81 KD cells (Figure 5K,L). Additionally, staining with Oil‐Red‐O confirmed the elevated lipid accumulation in GRP81 KD cells (60.87% ± 3.65% positive, Figure 5M,N). Interestingly, lipid associated protein PLIN2, which is known to coat lipid droplets in the skeletal muscle cells (Conte et al. 2013) was also upregulated with GPR81 KD (Figure 5O,P; Figure S3R,S). Collectively, these results suggest that GPR81 KD in young myoblasts leads to lipid accumulation as a result of increased lipid uptake and decreased mitochondrial fatty acid oxidation.

### 3.6 Loss of GPR81 Leads to Impaired Autophagy

**FIGURE 6:**
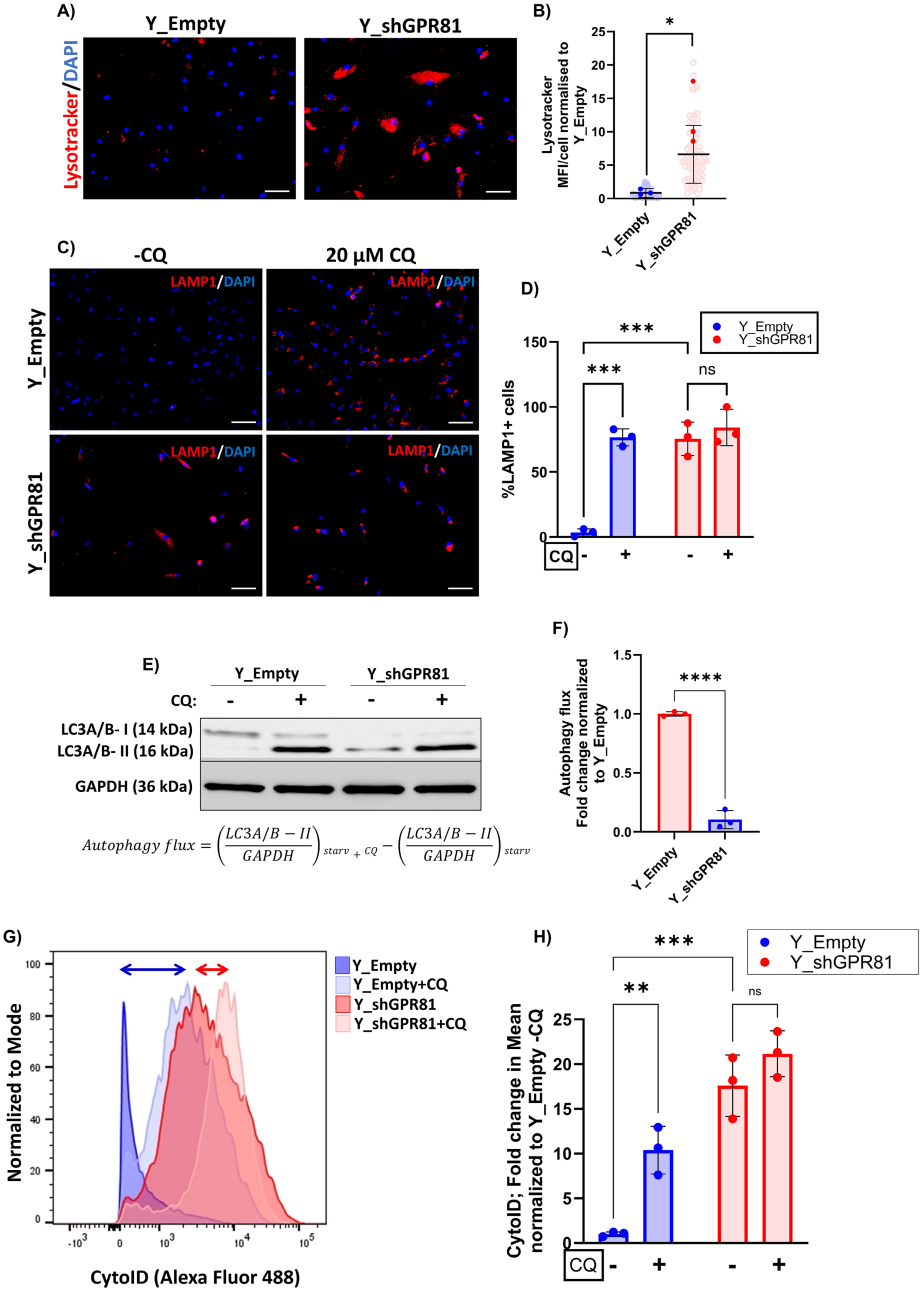
Knockdown of GPR81 in young myoblasts leads to impaired autophagy. (A) Representative images of Lysotracker (red) live staining in Y_Empty and Y_shGPR81 cells. (B) Quantification of Lysotracker intensity per cell, normalized to Y_Empty; data shown for > 100 cells from three independent biological replicates. (C) Immunostaining for LAMP1 (red) in Y_Empty and Y_shGPR81 myoblasts with and without overnight treatment with 20 μM CQ. (D) Quantification of percentage of LAMP1 positive cells; data shown for > 100 cells from three independent biological replicates. Nuclei for all images stained using Hoechst 33342 (blue). Scale bar (for A, C) represents 100 μm. (E) Western blots for LC3A/B protein upon starvation and starvation + CQ treatment for 6 h in Y_Empty and Y_shGPR81 cells. (F) Quantification of autophagy flux after normalization to GAPDH, using the formula: Autophagy flux = (LC3A/B‐II/GAPDH)_starv+CQ_ − (LC3A/B‐II/GAPDH)_starv_. (G) Flow cytometry histogram of cells stained with CYTO‐ID before or after starvation with CQ treatment. (H) Fold change in mean to CytoID signal normalized to Y_Empty (‐CQ). All data shown as mean ± SD. All experiments were performed using three independent biological replicates. *p < 0.05, **p < 0.01, ***p < 0.001, ****p < 0.0001.

Decreased rate of autophagy and accumulation of autophagic vesicles and lysosomes are hallmarks of the aged phenotype (Park et al. 2018; Narita et al. 2011). Therefore, we investigated whether accumulation of lipid droplets by GPR81 KD could be due to impaired autophagy. First, GPR81 KD led to increased lysosomal accumulation as evidenced by enhanced lysotracker staining (Figure 6A,B). We also assessed lysosomal trafficking by starving cells overnight while inhibiting autophagosome‐lysosome fusion by treatment with the lysosomal inhibitor chloroquine (CQ, 20 μM) and subsequently staining for the lysosomal protein LAMP1. Young cells transduced with empty vector did not stain for LAMP1; however, CQ treatment significantly enhanced LAMP1 staining (76.64 ± 8.84‐fold, Figure 6C,D). On the other hand, GPR81 KD cells had higher LAMP1 levels that did not increase further on CQ treatment, indicating impaired autophagy (Figure 6C,D).

To measure the autophagy flux, we performed western blots for LC3‐I/II after CQ treatment and observed higher LC3‐I/II accumulation in cells transduced with empty vector but not in GPR81 KD cells on CQ treatment after starvation, indicating impaired autophagy with loss of GPR81 (Figure 6E,F). These results were further validated with staining cells for the Cyto‐ID dye, which specifically binds to autophagic vacuoles and can be detected using flow cytometry. Myoblasts transduced with shGPR81 had greater Cyto‐ID intensity in basal conditions as compared to controls (empty vector), suggesting accumulated autophagosomes in these cells. Treatment with CQ resulted in a significantly greater shift in fluorescence intensity in Y_Empty cells as compared to Y_shGPR81 KD cells, indicating decreased autophagy flux upon loss of GPR81 (Figure 6G,H). These results confirm that knockdown of GPR81 disrupts autophagy, likely contributing to enhanced lipid accumulation in myoblasts.

### 3.7 GPR81 Agonists Alleviate Hallmarks of Senescence and Restore Myotube Differentiation

Since loss of GPR81 increased senescence hallmarks in myoblasts, we were interested to evaluate if agonists of GPR81 could alleviate markers of muscle aging. To this end, we treated senescent myoblasts with either 3‐Chloro‐5‐hydroxybenzoic acid (CHBA; 100 μM), GPR81 agonist 1 (Compound 2; C2; 5 μM) or Sodium L‐Lactate (10 mM) for 10 days in culture and assessed changes in senescence markers. Both CHBA and C2 have been used in the literature to selectively upregulate GPR81 activity (Dvorak et al. 2012; Sakurai et al. 2014). On the other hand, while lactate is the primary ligand of GPR81, it can also alter cellular activity by intracellular transport through monocarboxylate transporters such as MCT‐1 (Matsuhashi et al. 2025). Surprisingly, a 10‐day treatment of senescent myoblasts with CHBA, C2 or lactate increased GPR81 expression, with CHBA eliciting a greater response compared to C2 (Figure S4A–F).

**FIGURE 7:**
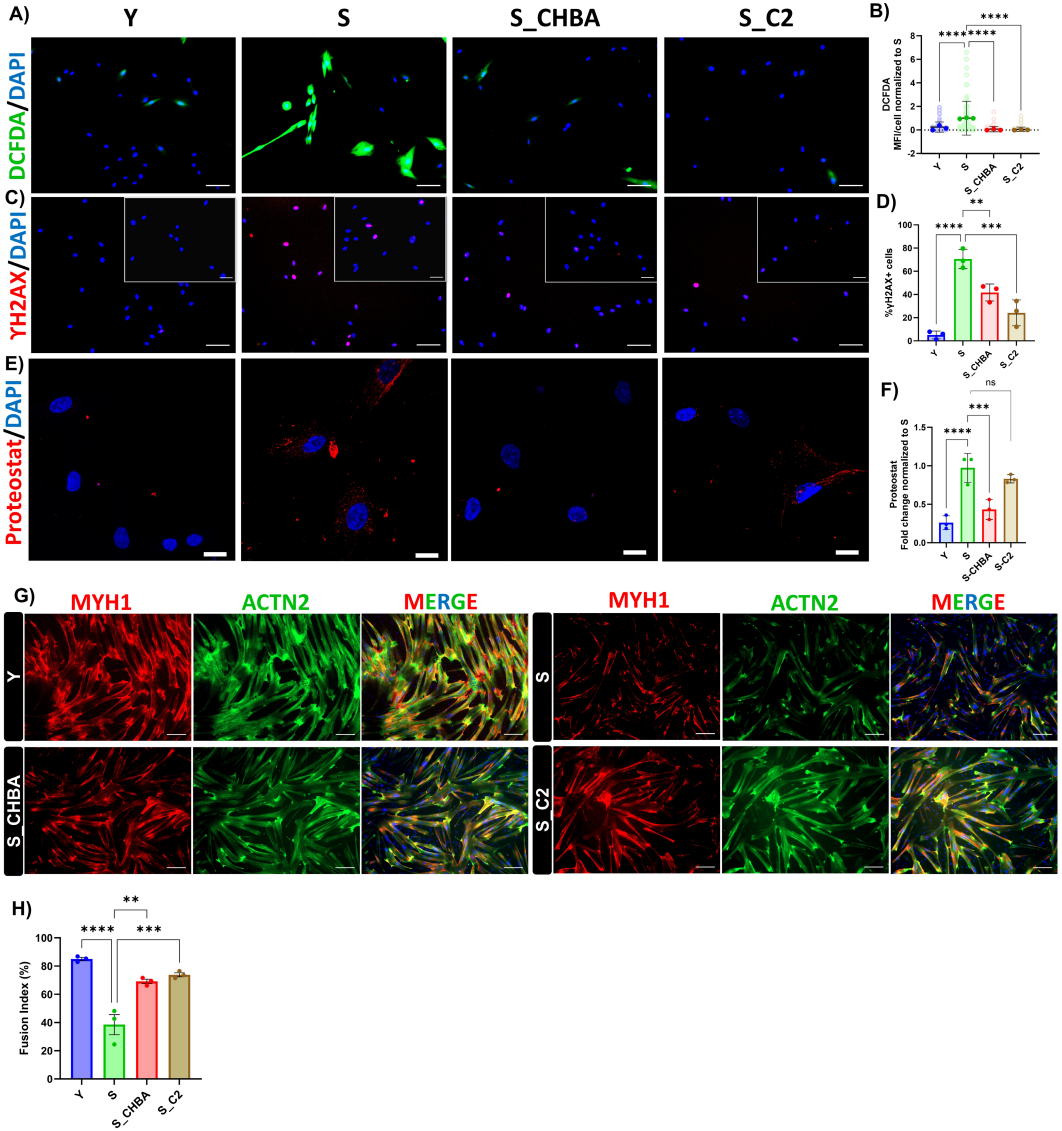
Treatment of senescent myoblasts with GPR81 agonists reverses markers of cellular senescence. (A) Representative images of DCFDA (green) live staining in Y, S, S_CHBA and S_C2 cells; scale bar represents 100 μm. (B) Quantification of DCFDA intensity per cell, normalized to S; data shown for > 100 cells from three independent biological replicates. (C) Immunostaining for phosphorylated form of histone H2AX (ϒ‐H2AX) (red) in Y, S, S_CHBA and S_C2 myoblasts; scale bar represents 100 μm; Insets at higher magnification with scale bar = 50 μm. (D) Quantification of percentage of ϒ‐H2AX positive cells. (E) Representative confocal images for Proteostat (red) staining to depict aggresome accumulation; scale bar represents 10 μm. (F) Quantification of fold change in number of proteostat positive cells, normalized to S. (G) Representative images of myoblasts differentiated to myotubes and stained for MYH1 (red) and ACTN2 (green); scale bar represents 200 μm. (H) Quantification for fusion index after myotube differentiation of Y, S, S_CHBA and S_C2 cells myoblasts. Nuclei for all images stained using Hoechst 33342 (blue). All data shown as mean ± SD for > 100 cells. All experiments were performed using three independent biological replicates. **p < 0.01, ***p < 0.001, ****p < 0.0001.

Treatment of senescent myoblasts with these agonists decreased several hallmarks of cellular senescence. Specifically, CHBA, C2 and lactate eliminated accumulation of ROS to similar levels as in young myoblasts (Figure 7A,B, Figure S4G,H) and reduced DNA damage, as evidenced by reduced fraction of cells positive for phosphorylated histone H2A.X (γH2AX; Figure 7C,D, Figure 4I,J). CHBA and lactate also decreased aggresome accumulation as shown by Proteostat staining, while C2 treatment did not have a significant effect (Figure 7E,F, Figure S4K,L). Surprisingly, GPR81 agonists enhanced the differentiation capacity of senescent myotubes, which is typically lost upon culture senescence. Treatment of senescent myoblasts with CHBA or C2 enhanced myotube formation as measured by the fusion index (Figure 7G,H) and expression of myosin heavy chain 1 (MYH1) and sarcomeric α‐Actinin (ACTN2) (MYH1+/ACTN2+ cells, Y: 85.04% ± 1.62%, S: 35.50% ± 10.09%, S_CHBA: 69.09% ± 2.24%, S_C2: 73.81% ± 2.03%). These results indicate that GPR81 agonists could reverse several aspects of skeletal muscle aging in vitro. Since lactate supplementation to media may have additional metabolic effects independent of GPR81 signaling, such as contribution to cellular bioenergetics via MCT‐1 mediate transport (Certo et al. 2022), most metabolic studies were performed using the selective GPR81 agonists‐ CHBA and C2.

### 3.8 GPR81 Agonists Improved Mitochondrial Membrane Potential and Decreased Intracellular Lipid Accumulation in Senescent Myoblasts

**FIGURE 8:**
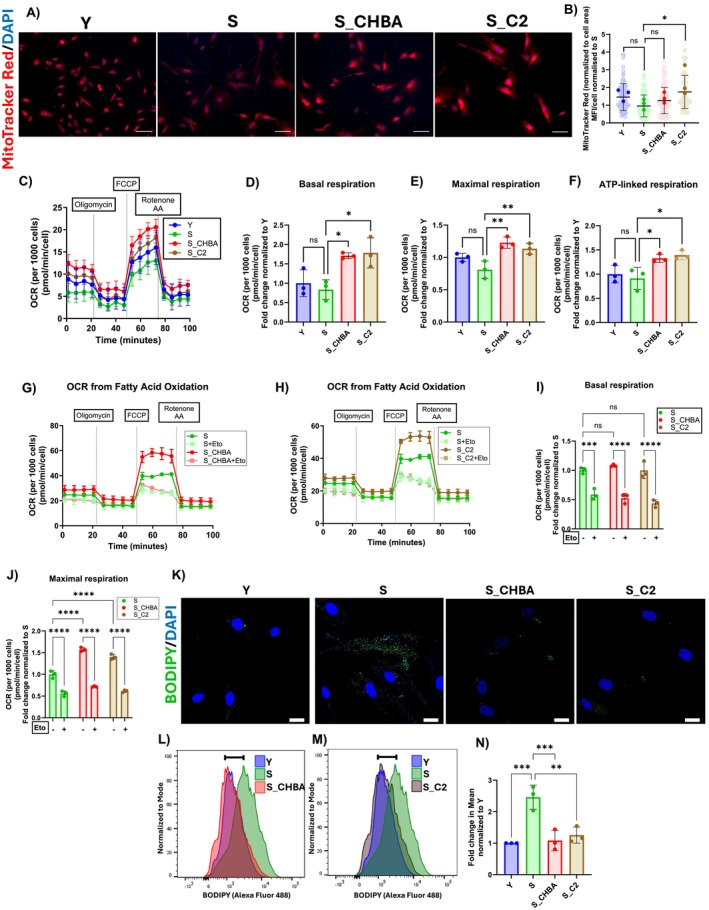
Treatment of senescent myoblasts with GPR81 agonists enhances lipid oxidation and reverses lipid accumulation. (A) Representative images of MitoTracker Red (red) live staining in Y, S, S_CHBA and S_C2 cells. Nuclei stained using Hoechst 33342 (blue). Scale bar represents 100 μm. (B) Quantification of MitoTracker Red intensity per cell, normalized to S and cell area; data shown for > 100 cells from three independent biological replicates. (C) Measurements of oxygen consumption rate (OCR) using Seahorse extracellular flux analyzer in Y, S, S_CHBA and S_C2 cells. (D) Basal Respiration, (E) Maximal Respiration and (F) ATP‐linked respiration calculated from OCR data in (C). OCR measured with or without etomoxir (Eto) inhibition in (G) S vs. S_CHBA and (H) S vs. S_C2 cells. (I) Basal Respiration and (J) Maximal Respiration calculated from OCR data in (G, H). (K) Representative confocal images for BODIPY (green) staining to depict lipid droplets in Y, S, S_CHBA and S_C2 myoblasts. Nuclei stained using Hoechst 33342 (blue). Scale bar represents 10 μm. Flow cytometry histograms for myoblasts stained with BODIPY (green) and detected using the Alexa Fluor 488 channel in (L) Y, S, S_CHBA and (M) Y, S, S_C2. (N) Fold change in mean BODIPY signal normalized to Y. All data shown as mean ± SD. All experiments were performed using three independent biological replicates. *p < 0.05, **p < 0.01, ***p < 0.001, ****p < 0.0001.

To evaluate the effect of GPR81 agonists on mitochondrial membrane potential, we stained senescent myoblasts for polarized (active) mitochondria after treatment with CHBA, C2 or lactate for 10 days. Both CHBA and C2 treatment showed trends of increased staining for MitoTracker Red, indicating restoration of mitochondrial membrane potential (Figure 8A,B; Y vs. S: *p*_s.c_. < 0.0001, *p*_b.r_. = ns; S vs. S_CHBA: *p*_s.c_. = 0.0129, *p*_b.r_. = ns; S vs. S_C2: *p*_s.c_. < 0.0001, *p*_b.r_. = 0.0409). Lactate treatment also showed increased MitoTracker Red staining (Figure S4M,N). To further confirm enhanced mitochondrial function, we measured OCR in young, senescent and senescent cells treated with CHBA or C2 using the Seahorse XF analyzer (Figure 8C). Indeed, both CHBA and C2 enhanced OCR and restored the levels of basal respiration (Figure 8D), maximal respiration (Figure 8E) and ATP‐linked respiration (Figure 8F) to the levels of young controls (OCR values were normalized to total mitochondrial content for each sample; Figure S4O).

Next, we evaluated the contribution of lipid oxidation to mitochondrial function in CHBA or C2 treated cells by treatment with etomoxir (Figure 8G,H). CHBA treatment significantly increased basal respiration (Figure 8I), and both CHBA and C2 increased maximal respiration that was inhibited by etomoxir to a greater extent than in untreated senescent cells (Figure 8J). In agreement, CHBA or C2 treated myoblasts showed decreased accumulation of lipid droplets, as evidenced by BODIPY staining (Figure 8K) and flow cytometry (CHBA: 1.69 ± 0.30‐fold decrease; C2: 1.17 ± 0.10‐fold decrease) (Figure 8L–N). These results demonstrate that GPR81 agonists enhanced mitochondrial respiration and decreased myosteatosis in senescent myoblasts.

### 3.9 Systemic Administration of CHBA in an Accelerated Mouse Model of Aging Altered the Muscle Transcriptome and Reversed Aging Hallmarks

Next, we evaluated whether the GPR81 agonist CHBA could alleviate senescence markers in vivo, using a mouse model of premature aging. Specifically, we employed Lamin A Knocked In (LAKI) mice that carry a mutation in the lamin A gene (lmnaG609G/G609G) resulting in accumulation of the truncated form of lamin A (progerin), which causes premature aging (Osorio et al. 2011). Mice carrying the homozygous mutation (LmnaG609G/G609G) have a very short lifespan of 3–4 months and exhibit accelerated onset of many age‐associated phenotypes, including weight loss, reduced activity, significantly decreased muscle mass, and impaired muscle regeneration (Zaghini et al. 2020).

**FIGURE 9:**
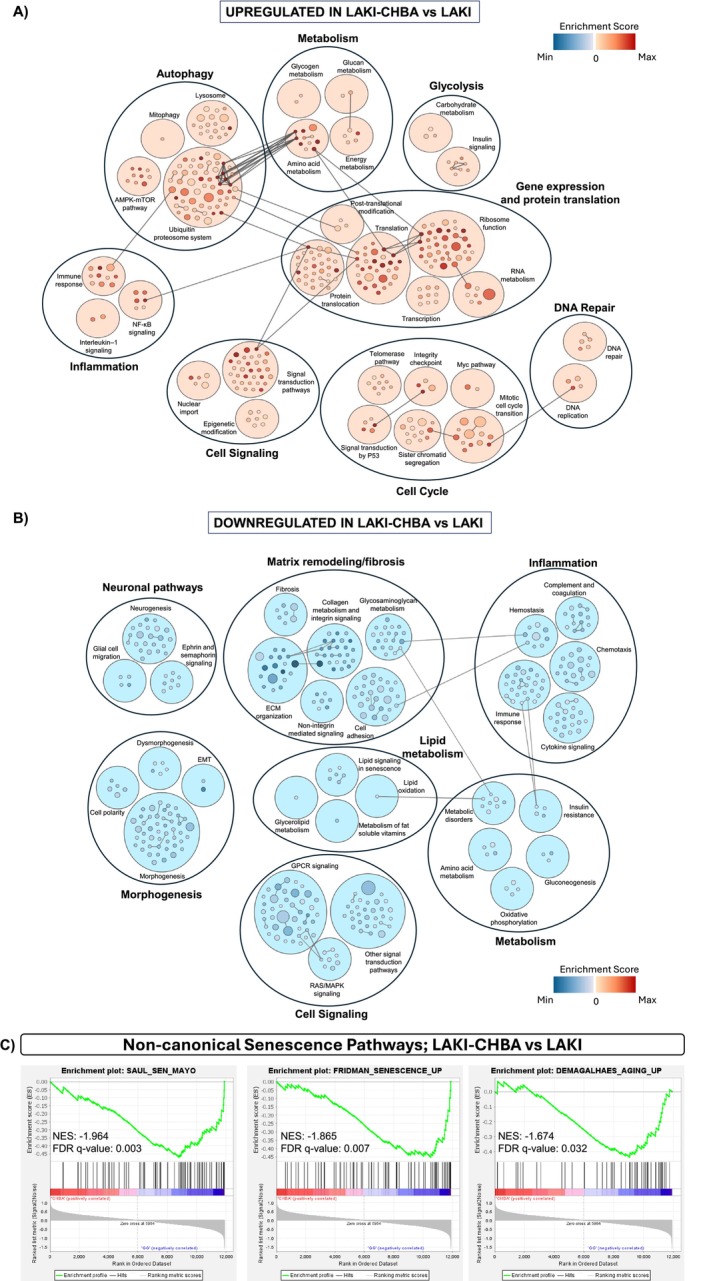
CHBA administration in progeric mice altered muscle transcriptome and reversed aging hallmarks. Cytoscape networks showing top 200 GSEA pathways that were (A) upregulated and (B) downregulated in TA muscles of LAKI‐CHBA mice, as compared to LAKI mice. Lines depict the transcriptional similarity in genes within each pathway cluster. Node size represents the number of genes identified in each gene set. Node color represents the degree of enrichment based on enrichment scores. (C) Enrichment plots showing senescence‐associated pathways downregulated in muscle of LAKI‐CHBA as compared to LAKI mice; *n* = 7 biological replicates for both LAKI‐CHBA and LAKI mice.

Homozygous LmnaG609G/G609G mice received intraperitoneal (I.P.) injections of CHBA (LAKI‐CHBA) at a dose of 10 mg/kg. Injections began when the mice were 10 weeks old and continued for five weeks, with three injections per week. Mice that received only the vehicle (DMSO in corn oil) served as controls (LAKI, Figure S5A). The dosage and duration of CHBA treatment was determined based on previously published studies (Sakurai et al. 2014; Li et al. 2022; Ohno et al. 2023), and I.P. was chosen instead of oral gavage to enhance bioavailability and minimize procedural stress and potential mortality. Similar to our in vitro findings, we found that I.P. administration of CHBA for 5 weeks enhanced GPR81 expression in muscle tissue, though no difference in GPR81 was found in WT and LAKI mice (Figure S5B,C). Next, muscles were isolated from LAKI‐CHBA and LAKI mice and global transcriptional changes were assessed through RNA sequencing. Pathway enrichment analysis revealed that CHBA treatment significantly upregulated several pathways including those involved in cellular functions known to be downregulated with age (Moiseeva et al. 2023) such as autophagy, energy metabolism, glycolysis, gene expression and protein translation, DNA repair, cell cycle and signaling (Figure 9A). In parallel, pathways pertaining to muscle fibrosis, metabolic dysfunction, inflammation and lipid metabolism were downregulated with CHBA treatment (Figure 9B). CHBA treatment also decreased expression of senescence‐associated genes contributing to senescence across tissues and species such as the SAUL_SEN_MAYO gene set (Saul et al. 2022); regulator genes primarily affecting pRB/p53, cytoskeletal, interferon‐related, insulin growth factor‐related, MAP kinase and oxidative stress pathways as reported in the FRIDMAN_SENESCENCE_UP gene set (Fridman and Tainsky 2008); and genes affected by aging in mice, rats and humans, as reported in the DEMAGALHAES_AGING_UP dataset (De Magalhães et al. 2009) (Figure 9C). These results indicate that systemic CHBA administration in progeric mice rewired their transcriptome leading to alleviation of aging hallmarks.

### 3.10 CHBA Administration in Progeria Mice Improved Mouse Health by Decreasing Intramyocellular Lipid Accumulation

**FIGURE 10:**
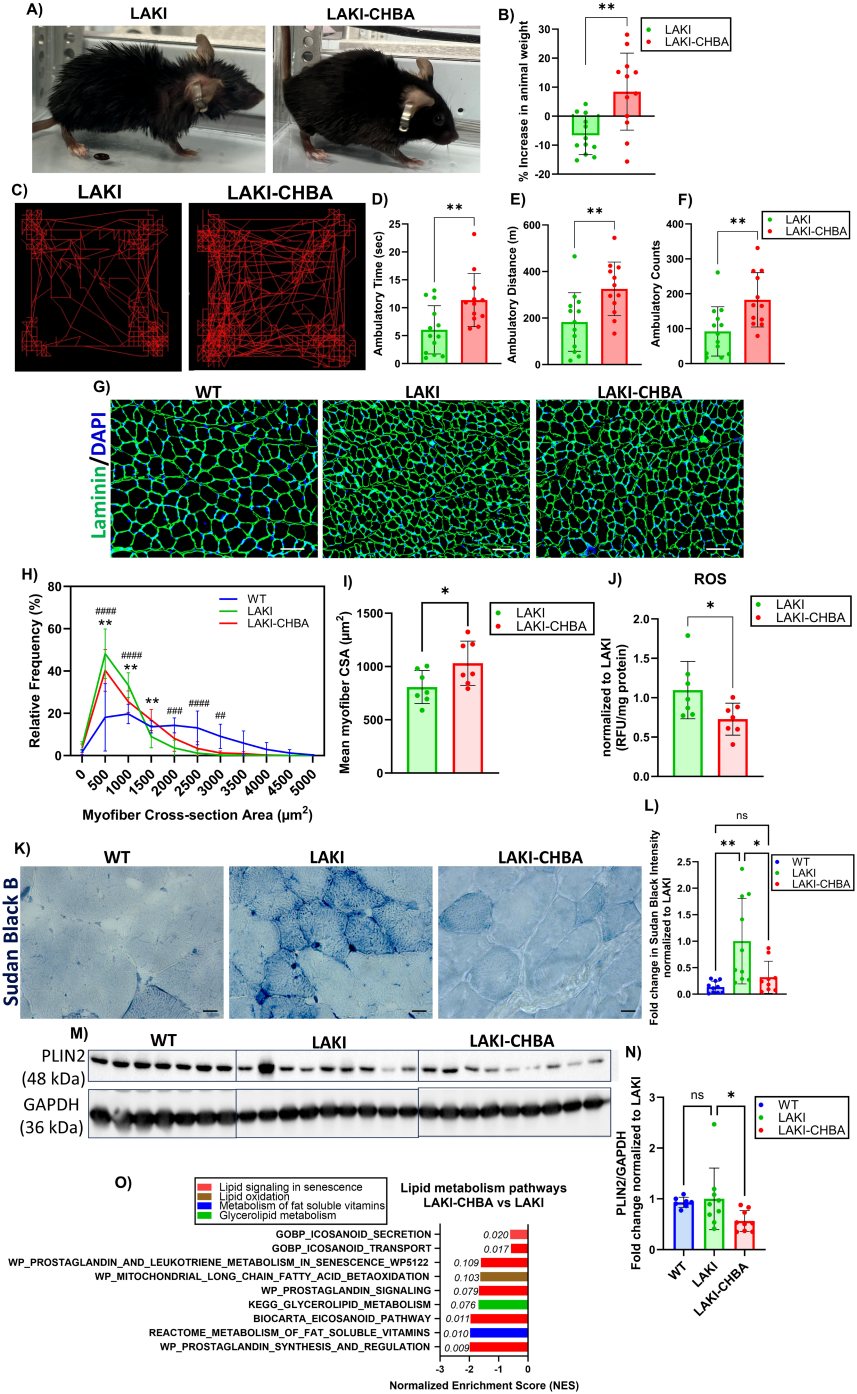
Administration of CHBA improves mouse health and decreases intramyocellular lipids in skeletal muscle of progeric mice. (A) Representative images of mice after 5 weeks of Vehicle (LAKI) or CHBA (LAKI‐CHBA) administration. (B) Quantification of percent increase in animal weight in LAKI and LAKI‐CHBA mice; *n* = 12 biological replicates each. (C) Representative paths (in red) traveled by LAKI and LAKI‐CHBA animals during open field activity test. (D) ambulatory time, (E) ambulatory distance and (F) ambulatory counts calculated in open field activity test; LAKI: *N* = 13, LAKI‐CHBA: *N* = 12 biological replicates. (G) Representative images of TA muscle fiber size depicted using Laminin (green) staining. Nuclei stained using Hoechst 33342 (blue). Scale bar represents 100 μm. (H) Frequency distribution of myofiber CSA in TA muscle from WT, LAKI, LAKI‐CHBA mice 5 weeks after Vehicle/CHBA administration (*Represents statistically significant difference in fiber size in LAKI and LAKI‐CHBA mice, ^#^Represents statistically significant difference in fiber size in WT and LAKI mice); *n* = 7 biological replicates each. (I) Mean myofiber CSA in TA muscle from LAKI and LAKI‐CHBA mice; *n* = 7 biological replicates each. (J) Measurement of ROS levels in GA muscle in LAKI and LAKI‐CHBA animals, normalized to LAKI; *n* = 7 biological replicates each. (K) Representative images of staining for Sudan Black B in TA muscles from WT, LAKI and LAKI‐CHBA mice. Scale bar represents 10 μm. (L) Quantification of fold change in Sudan Black B intensity, normalized to LAKI; WT and LAKI: *N* = 10 each, LAKI‐CHBA: *N* = 9 biological replicates. (M) Western blot for PLIN2 in GA muscle isolated from WT, LAKI and LAKI‐CHBA mice, and (N) quantification of PLIN2 expression level after normalization to GAPDH; WT: *N* = 7, LAKI and LAKI‐CHBA: *N* = 9 biological replicates each. (O) GSEA enrichment analysis depicting lipid metabolism associated pathways downregulated by CHBA treatment in LAKI mice. FDR *q*‐values for each pathway are depicted next to bars; *n* = 7 biological replicates for both LAKI‐CHBA and LAKI mice. All data shown as mean ± SD. *p < 0.05, **p < 0.01.

We next examined the effects of CHBA administration on mouse health, muscle structure, and lipid metabolism. We found that control LAKI mice that received vehicle for 5 weeks appeared weak and frail and had an unkept coat, signs of poor health. On the other hand, mice treated with CHBA did not show these aging traits and appeared well‐groomed (Figure 10A). CHBA administration led to an increase in body weight (~8.45% increase), while control LAKI mice kept losing weight despite daily supportive care (~6.60% decrease; Figure 10B). To evaluate the motor activity and exploratory drive of animals, we performed the open field activity test, which serves as an indicator of mouse locomotor activity and anxiety, with decreased activity commonly correlating with aging (Shoji et al. 2016). Mice were placed in an open arena and allowed to explore the cage for 30 min while their movement was recorded in real time (Figure S6A). Baseline measurements recorded before the start of CHBA/Vehicle administration demonstrated that all animals performed equally well with no significant difference in time of movement (ambulatory time; Figure S6B), distance traveled (ambulatory distance, Figure S6C), and steps taken (ambulatory counts, Figure S6D). Interestingly, 5 weeks later, locomotor activity decreased, albeit to a lesser extent in CHBA‐treated mice (Figure 10C), which exhibited increased ambulatory time, distance, and counts when compared to untreated mice (Figure 10D–F).

Next, we isolated the TA muscle from LAKI and LAKI‐CHBA mice and assessed changes in muscle structure and function. Measurements of muscle fiber size revealed that CHBA administration resulted in an increase in myofiber area, reducing the number of small fibers in the 250–1250 μm^2^ range, while increasing the number of larger fibers in the 1250–1750 μm^2^ range (Figure 10G,H). The mean myofiber cross section area (CSA) was also higher in TA muscle isolated from LAKI‐CHBA mice (Figure 10I). Next, because our in vitro experiments revealed changes in mitochondrial membrane potential and respiration, we assessed the relative levels of oxidative phosphorylation (OXPHOS) complexes I–V; NDUFB8, SDHB, UQCRC2, MTCO1, and ATP5A in protein isolated from the TA muscle (Figure S6E). Interestingly, CHBA administration did not alter the abundance of any of these complexes (Figure S6F–J). Total mitochondrial content, measured via citrate synthase activity, also remained unchanged between LAKI and LAKI‐CHBA mice (Figure S6K). Nevertheless, consistent with our in vitro findings, we observed a reduction in ROS accumulation in GA muscles of LAKI‐CHBA mice (Figure 10J). Staining muscle sections with the lipophilic dye Sudan Black B showed that CHBA treatment decreased the intramyocellular lipid content of LAKI muscle to levels similar to those found in young WT muscle (Figure 10K,L; Figure S6L). In addition, CHBA treatment significantly reduced expression of the lipid‐droplet associated protein, PLIN2 in TA muscle (Figure 10M,N). Consistent with this, RNA sequencing revealed a decrease in lipid signaling and metabolism pathways in CHBA‐treated muscle. Gene Set Enrichment Analysis (GSEA) further confirmed the downregulation of multiple pathways related to lipid signaling, lipid oxidation, metabolism of fat‐soluble vitamins and glycerolipid metabolism in LAKI‐CHBA mice (Figure 10O). These results demonstrate that CHBA administration led to an overall improvement in health and a reduction in lipid accumulation in progeroid mice.

### 3.11 CHBA Administration Enhanced Muscle Regeneration in Progeroid Model of Accelerated Aging

**FIGURE 11:**
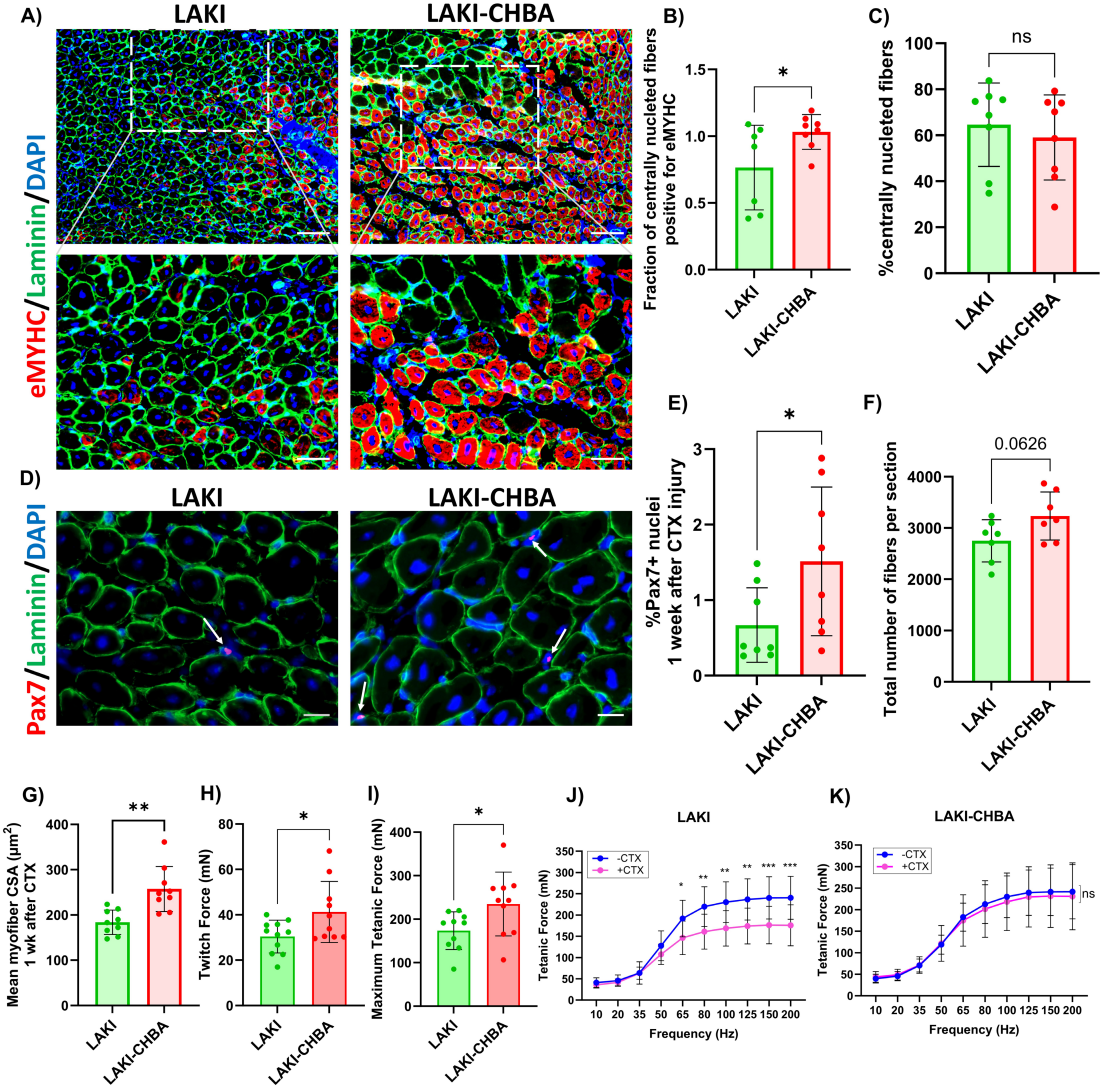
Administration of CHBA improves muscle regeneration in progeric mice. (A) Immunostaining for Laminin (green) and eMYHC (red) in LAKI and LAKI‐CHBA mice 1 week after CTX injury. Nuclei stained using Hoechst 33342 (blue). Scale bar represents 100 μm. Insets are at higher magnification with scale bar 50 μm. (B) Quantification of fraction of centrally nucleated fibers that stain for eMYHC. (C) Quantification of percentage of centrally nucleated myofibers; *n* = 8 biological replicates each. (D) Immunostaining for Laminin (green) and Pax7 (red) in LAKI and LAKI‐CHBA mice 1 week after CTX injury. Nuclei stained using Hoechst 33342 (blue). Scale bar represents 10 μm. (E) Quantification of PAX7+ nuclei normalized to total nuclei; *n* = 8 biological replicates each. (F) Quantification of total number of myofibers in each TA muscle section of LAKI and LAKI‐CHBA mice; *n* = 7 biological replicates each. (G) Mean myofiber CSA in TA muscle from LAKI and LAKI‐CHBA mice 1 week after CTX injury; *n* = 9 biological replicates each. (H) Twitch force recorded in CTX‐injured leg of LAKI and LAKI‐CHBA mice 1 week after injury. (I) Maximum tetanic force recorded at stimulation frequency of 150 Hz in CTX‐injured leg of LAKI and LAKI‐CHBA mice 1 week after injury. (J) Force frequency curve in LAKI and (K) LAKI‐CHBA mice before and after CTX injury; LAKI: *N* = 11; LAKI‐CHBA: *N* = 10 biological replicates. All data shown as mean ± SD. *p < 0.05, **p < 0.01, ***p < 0.001.

One of the key hallmarks of muscle aging is its impaired ability to regenerate after injury. To investigate if CHBA administration could enhance muscle regeneration, we utilized cardiotoxin (CTX) to induce injury in LAKI and LAKI‐CHBA mice. To this end, LAKI mice were treated with either vehicle (LAKI) or CHBA (LAKI‐CHBA) for 4 weeks. Their right TA muscle was then subjected to CTX injury and allowed to regenerate for a week, with continued drug administration. At that time, we measured the muscle force generation capacity before harvesting the TA muscle for histological evaluation of tissue regeneration (Figure S7A). We first quantified the number of myofibers that were positive for embryonic myosin heavy chain (eMYHC), an indicator of regenerating fibers. Interestingly, CHBA treatment significantly increased the fraction of myofibers that were both centrally nucleated and positive for eMYHC (Figure 11A,B), though total fibers that were multinucleated did not change with treatment (Figure 11C). Notably, the number of PAX7+ satellite cells increased (Figure 11D,E). The total number of myofibers showed an increasing trend with CHBA treatment, though the increase was not statistically significant (Figure 11F). We also observed an increase in myofiber CSA in TA muscle from LAKI‐CHBA mice, confirming enhanced muscle regeneration (Figure 11G).

To assess the effect of CHBA on muscle function, we measured the aggregate torque produced by the dorsiflexor muscles of live mice using a force transducer (Figure S7B). Specifically, we measured twitch force, maximum isometric tetanic force, and force–frequency relationship generated by the right limb, which received CTX, while the left limb served as control. First, we measured these forces before the start of treatment (baseline) and found no significant differences in twitch force, maximum tetanic force or force at different stimulation frequencies between LAKI and LAKI‐CHBA groups (Figure S7C–E). At 1 week after CTX injury (5 weeks CHBA treatment), the injured leg of CHBA‐treated animals generated greater twitch force (Figure 11H) and greater maximum tetanic force (Figure 11I) as compared to untreated LAKI controls. Both twitch and tetanic forces were restored to pre‐injury levels, when treated with CHBA (Figure S7F,G). Furthermore, in contrast to LAKI controls, LAKI‐CHBA mice fully restored muscle force to comparable levels as non‐injured muscle at high stimulation frequencies (Figure 11J,K), similar to their young WT counterparts (Figure S7H). These findings suggest improved recovery of muscle function following injury with CHBA treatment. Taken together, these results indicate that CHBA treatment enhances muscle regeneration and functional recovery after CTX injury.

## 4 Discussion

In this study, we implicate the lactate receptor GPR81 in age‐associated loss of mitochondrial function and lipid accumulation in skeletal muscle cells. Skeletal muscle plays an important role in carbohydrate and lipid metabolism and closely interacts with the liver and adipose tissue to maintain metabolic homeostasis (Shimizu et al. 2015; da Silva Rosa et al. 2020). Lipids play a key role in the function of each of these organs, and cellular stress induced by accumulation of lipid intermediates such as diacylglycerols (DAGs), ceramides, and triglycerides has been shown to facilitate the development of insulin resistance in the muscle (Erion and Shulman 2010). Further, skeletal muscle aging is associated with decreased mitochondrial function, leading to reduced muscle oxidative capacity, including lipid oxidation, further exacerbating lipotoxicity (Conley et al. 2000; Crane et al. 2010). In the present study, we focus on the replicative senescence model of aging using myoblasts from biological donors of different ages. While we did not observe a systematic influence of donor age on the onset of replicative senescence or on the experimental readouts assessed under our in vitro conditions, the effects of culture‐driven replicative senescence may dominate over donor age‐associated variability, which remains unexplored in this study.

We report that the expression of key enzymes in the lipid oxidation and mobilization pathways is altered in senescent myoblasts. Specifically, the fatty acid transporter, CD36, which was previously implicated in regulating production of senescence‐associated secretory phenotype (SASP) (Chong et al. 2018; Saitou et al. 2018), was upregulated with aging. Despite increased lipid uptake, senescent myoblasts exhibited loss of respiratory capacity and lipid oxidation. These results are in agreement with previously published data that established stark differences in lipid and mitochondrial structure and dynamics in young vs. old individuals. Biopsies of skeletal muscle from old human donors contained higher density and larger intramyocellular lipids (IMCL) and reduced number of mitochondria as compared to young donors (Crane et al. 2010). On the other hand, physical activity was shown to protect aged muscle from lipid accumulation and deterioration of mitochondria (St‐Jean‐Pelletier et al. 2017). Since high intensity exercise is associated with accumulation of lactate in the skeletal muscle (Pilegaard et al. 1999), we hypothesized that lactate receptor signaling might play a key role in skeletal muscle lipid oxidation.

The lactate receptor GPR81 has been shown to inhibit lipolysis in the adipose tissue (Liu et al. 2009; Cai et al. 2008). Although GPR81 is also widely expressed in skeletal muscle (Nordström et al. 2023), its role in the context of lipid metabolism and muscle aging remains unknown. Interestingly, we found that GPR81 expression diminished in biologically aged muscle. Moreover, the loss of GPR81 in young myoblasts led to an increase in key hallmarks of aging, including DNA damage, cell cycle arrest, ROS accumulation, and impaired differentiation into myotubes. Surprisingly, loss of GPR81 in human myoblasts was associated with accumulation of lipid droplets (LD), as seen by staining with BODIPY and Oil‐Red‐O and increased staining for the skeletal muscle specific perilipin protein, PLIN2, which is responsible for IMCL synthesis and LD growth (Morales et al. 2017). These results suggest that the role of GPR81 in lipid metabolism of skeletal muscle may be opposite to that of adipose tissue. In adipose tissue, lactate binding to GPR81 activates cyclic AMP (cAMP)‐protein kinase (PKA)‐cAMP‐response element binding (CREB) signaling leading to inhibition of lipolysis (Nordström et al. 2023). Conversely, our data show that GPR81 activation in skeletal muscle promotes lipid breakdown and may play a role in preventing age‐related lipotoxicity. In agreement with our findings, Wu et al. have reported that loss of GPR81 expression in the liver led to enhanced hepatic lipid accumulation (Wu et al. 2022). These results are novel, as the role of GPR81 in skeletal muscle lipid metabolism has not been previously investigated. However, lactate binding to GPR81 does not regulate the cAMP‐PKA‐CREB signaling pathway in muscle, suggesting that an alternative mechanism may be at work (Nordström et al. 2023).

Lipids in skeletal muscle are primarily catabolized in the mitochondria through β‐oxidation to generate energy or are degraded during the process of autophagy. We have previously published that skeletal muscle aging leads to impaired mitochondrial function and autophagy (Shahini et al. 2021). In this study, we demonstrate that GPR81 plays a key role in mitochondrial function, as GPR81 knockdown decreased OCR, led to accumulation of lysosomes, and decreased autophagy flux. As a result, lipids taken up by senescent myoblasts are not degraded via mitochondrial oxidation or autophagy and instead accumulate as lipotoxic waste, further accelerating the process of aging.

These results prompted us to evaluate whether GPR81 agonists could ameliorate markers of muscle aging by inducing lipolysis. To this end, we treated senescent human myoblasts with previously published GPR81 agonists, CHBA and C2. CHBA has been widely recognized as a selective agonist of GPR81 and is well‐tolerated in vivo (Dvorak et al. 2012; Li et al. 2022; Engelstoft et al. 2013). More recently, the small molecule C2 was established as a potent and selective GPR81 agonist, but its in vivo efficacy is yet to be established (Sakurai et al. 2014). We limited most of our study to the use of CHBA and C2, and not lactate, since lactate is known to affect other nonspecific signaling pathways, such as affecting bioenergetics through cellular uptake via MCT‐1 (Matsuhashi et al. 2025; Certo et al. 2022; Tsukamoto et al. 2018). Interestingly, treatment of senescent human myoblasts with either GPR81 agonist could reverse several aging hallmarks, including ROS accumulation, DNA damage, and aggresome accumulation. Furthermore, all agonists improved mitochondrial membrane potential to levels comparable to young myoblasts, as indicated by increased staining with MitoTracker Red and enhanced oxygen consumption (OCR). Improved mitochondrial oxidation enhanced lipid breakdown, thereby reducing lipid accumulation in senescent myoblasts. Therefore, both CHBA and C2 are promising candidates for reversing age‐associated myosteatosis in human myoblasts in culture.

While increased accumulation of lipid intermediates is known to occur in naturally aged muscle, we also found that lipid metabolism is impaired in fast aging models, such as the Hutchinson–Gilford Progeria Syndrome (HGPS) or progeria mice. In this context, we found that GPR81 expression is not significantly altered in LAKI mice, suggesting that alterations in GPR81 signaling, rather than expression, are the defining feature of this model of accelerated aging. It is possible that cell‐ or fiber type‐specific differences in GPR81 expression between WT and LAKI mice could contribute to the lack of detectable change in bulk tissue measurements; however, this remains to be determined. Notably, systemic administration of CHBA to LAKI mice enhanced GPR81 expression and improved their healthspan dramatically. Control untreated LAKI mice exhibited continuous decline in health and activity throughout the period of our study, despite regular supportive care. In contrast, CHBA‐treated LAKI mice looked healthier and displayed increased activity. While we did not observe any change in muscle weights of LAKI or LAKI‐CHBA mice, the body weight of CHBA‐treated animals increased significantly. An increase in body weight of progeric animals is often associated with increased lifespan (Kreienkamp et al. 2019), but the effect of CHBA on lifespan of LAKI mice is yet to be established. Regardless, we observed an increase in skeletal muscle fiber size, indicating reduced atrophy following CHBA treatment. Moreover, CHBA administration also led to decreased IMCLs, almost to the level of young healthy controls, and significantly decreased ROS accumulation in LAKI mice. RNA sequencing analysis in muscles from CHBA treated mice revealed a downregulation in pathways associated with lipid oxidation. A possible explanation to this could be that CHBA treatment reduces myocellular lipid accumulation, thereby lowering the requirement for lipid breakdown after prolonged treatment, and may reflect the long‐term metabolic adaptation of the muscle to CHBA treatment. Importantly, while no changes in mitochondrial content or OXPHOS subunit abundance were observed in LAKI‐CHBA mice, these measurements do not capture mitochondrial respiratory function. Thus, potential alterations in mitochondrial efficiency, coupling, or bioenergetic capacity remain possible and will require direct functional assessment in future studies. Collectively, our results suggest that systemic administration of CHBA prevented progression of age‐associated lipotoxicity and myosteatosis.

Previously, it was established that lipid droplet dynamics play a key role in the function of skeletal muscle satellite cells, and hence skeletal muscle regeneration. Satellite cells with low levels of lipid droplet accumulation enhance the muscle's regenerative capacity, whereas elevated lipid droplet accumulation impairs muscle regeneration (Yue et al. 2022; Akhmedov and Berdeaux 2013) and diminishes muscle strength and function (Hilton et al. 2008). Interestingly, while muscle healing capacity is impaired in progeria mice, CHBA treatment significantly enhanced muscle regeneration after CTX injury, as evidenced by the significantly increased number of Pax7+ satellite cells and eMYHC+ regenerating myofibers. Most notably, CHBA treatment restored the ability of muscle to generate force, as shown by isometric force measurements of the dorsiflexor muscles following electrical stimulation. Collectively, these results indicate that activating the lactate receptor by CHBA reversed age‐associated loss of muscle strength and function, possibly by reversing age‐associated myosteatosis. Future work will aim to extend these findings from progeroid models of aging to naturally aged mice to evaluate whether CHBA may have potential as a therapeutic strategy for sarcopenia in physiologically relevant preclinical models of aging.

While our study identifies a novel role for GPR81 in regulating lipid metabolism in cellular and progeroid models of muscle aging, several mechanistic questions remain to be addressed. Future work will focus on defining how activation of GPR81 modulates cellular senescence in naturally aged mice, including through genetic gain‐of‐function approaches. Reduced GPR81 expression in senescent myoblasts may be secondary to decreased receptor activation rather than primary transcriptional downregulation. Supporting this possibility, our previous work has shown that glycolytic activity and extracellular lactate levels are reduced in senescent human and mouse myoblasts (Rajabian, Choudhury, et al. 2023; Rajabian, Ikhapoh, et al. 2023). Since lactate is the endogenous ligand for GPR81, reduced lactate production during senescence could lead to diminished receptor activation and downstream signaling. This is supported by our findings that pharmacological activation of GPR81 could restore the metabolic function in senescent myoblasts. Further investigation into how glycolytic metabolism is altered upon loss of GPR81 may also provide broader insight into how loss of mitochondrial activity is integrated with shifts in cellular metabolic pathways during senescence.

Our study relies on Mito stress test as a readout for mitochondrial activity and function. While this assay provides valuable insights into mitochondrial function, several limitations should be considered when interpreting the data. A key limitation of this assay is its reliance on sequential pharmacological injections within the same well, which progressively disrupt cellular physiology. The cumulative effects of oligomycin, uncouplers, and electron transport chain inhibitors can alter redox balance, substrate utilization, and membrane potential, making later measurements difficult to interpret. Further, the assay readouts, including basal respiration, ATP‐linked respiration, and maximal respiration, can be influenced by factors such as uncoupler dose, cell density, energy substrate availability, and normalization methods. The data has been normalized to the cell number to account for differences in proliferation of cells after GPR81 knockdown or treatment with agonists prior to the assay. OCR values have also been normalized to mitochondrial content (mtDNA/nDNA) to account for differences in mitochondrial abundance between groups. However, mitochondrial content measurements reflect total mitochondria, which may include active and inactive ones, complicating the interpretation. Alternative proxy measurements of mitochondrial content, such as CS activity and OxPhos complex abundance, may provide more clarity.

In conclusion, we report that the lactate receptor GPR81 plays a critical role in maintaining skeletal muscle health and function in cellular and progeroid models of muscle aging. Loss of GPR81 leads to impaired mitochondrial membrane potential and respiration and impaired autophagy, causing accumulation of lipid droplets in myoblasts. Conversely, activating GPR81 signaling via chemical agonists, such as CHBA, improved mitochondrial function, decreased lipid accumulation, and enhanced skeletal muscle regeneration in progeric mice. Interestingly, the mechanism by which GPR81 influences lipid metabolism in skeletal muscle may differ from its role in adipose tissue, highlighting the need for further studies to elucidate how GPR81 signaling regulates lipid oxidation in muscle.

## Author Contributions

Experiments were planned and designed by P.M. and S.T.A. All in vitro experimental data were generated and collected by P.M., S.H.B., J.T., M.E. and D.C. All in vivo experiments were done by P.M., S.H.B., and P.L. Data analysis and interpretation involved P.M., S.H.B, J.T., S.C., G.E.A.‐G. and S.T.A. RNA sequencing analysis was done by Y.Z., J.W., S.L., S.H.B. and P.M. Writing and critical revisions of the manuscript were performed by P.M., P.L. and S.T.A.

## Funding

This work was supported by a grant from the National Institutes of Health, R01AG068250 to S.T.A.

## Conflicts of Interest

The authors declare no conflicts of interest.

## Supporting information


**Figure S1:** (A) Flow cytometry histograms for myoblasts stained with BODIPY (green) and detected using Alexa Fluor 488 channel. (B) Quantitative real‐time PCR quantification of mtDNA relative to nDNA (mtDNA/nDNA) as measured by primers specific to mitochondrial gene *MT‐TL1* and nuclear gene *β2M*. (C) Western blot for OxPhos complexes I‐V in Y and S myoblasts. Quantification was performed after normalization to GAPDH for (D) Complex V‐ ATP5A (E) Complex III‐ UQCRC2 (F) Complex II‐ SDHB (G) Complex IV‐ COXII and (H) Complex I‐ NDUFB8. All data shown as mean ± SD. All experiments were performed using three independent biological replicates. **p < 0.01.
**Figure S2:** (A) Western blots for GPR81 protein after knockdown with shGPR81 (Sigma) vector, quantified in (B) after normalization to GAPDH. (C) Quantitative real‐time PCR for *GPR81* internally normalized to *RPL32* cycle number followed by normalization to Y_Empty. (D) Western blots for GPR81 protein after knockdown with shGPR81 (shLVDP) vector, quantified in (E) after normalization to GAPDH. (F) Senescence‐associated β‐galactosidase (SA‐β‐Gal) staining in Y_Empty, Y_KD1 and Y_KD2 cells. Scale bar represents 100 μm. (G) Quantification for percentage of SA‐β‐Gal cells. (H) Representative images of DCFDA (green) live staining. Scale bar represents 100 μm. (I) Quantification of DCFDA intensity per cell for > 150 cells from three independent biological replicates, normalized to Y_Empty. (J) Immunostaining for phosphorylated form of histone H2AX (ϒ‐H2AX) (red) in Y_Empty, Y_KD1 and Y_KD2 myoblasts. Scale bar represents 100 μm; Insets at higher magnification with scale bar 50 μm. (K) Quantification of percentage of ϒ‐H2AX positive cells. (L) Immunostaining for P21 (red) in Y_Empty, Y_KD1 and Y_KD2 myoblasts; Insets at higher magnification with scale bar 50 μm. (M) Quantification of percentage of P21 positive cells, normalized to Y_Empty. (N) Representative confocal images for Proteostat (red) staining to depict aggresome accumulation in Y_Empty, Y_KD1 and Y_KD2 myoblasts. Scale bar represents 10 μm. (O) Quantification of proteostat intensity per cell, normalized to Y_Empty (P) Representative images of myoblasts differentiated to myotubes and stained for MYH1 (red) and ACTN2 (green); scale bar represents 200 μm. (Q) Quantification for fusion index after myotube differentiation of Y_Empty, Y_KD1 and Y_KD2 myoblasts. Nuclei for all images stained using Hoechst 33342 (blue). (R) Quantitative real‐time PCR for myogenic genes *PAX7*, *MYF5*, *DES*, *MRF4* and *MEF2C*, internally normalized to *RPL32* cycle number followed by normalization to Y_Empty. All data shown as mean ± SD. All experiments were performed using three independent biological replicates. *p < 0.05, **p < 0.01, ***p < 0.001, ****p < 0.0001.
**Figure S3:** (A, B) Representative images and quantification of mitotracker red (red), (C, D) mitotracker green (green) and (E) merged images of mitotracker red and green live staining in Y_Empty, Y_KD1 and Y_KD2 cells. Quantifications depict intensity per cell normalized to Y_Empty; data shown for > 100 cells from three independent biological replicates. (F) Quantification for ratio of mitotracker red to green signal for each cell, normalized to Y_Empty; data shown for > 100 cells from three independent biological replicates. Nuclei for all images stained using Hoechst 33342 (blue). Scale bar (for A, C, E) represents 100 μm. (G) Quantitative real‐time PCR to assess the level of mtDNA relative to nDNA (mtDNA/nDNA) using primers specific to mitochondrial gene *MT‐TL1* and nuclear gene *β2M*. (H) Measurements of oxygen consumption rate (OCR) using Seahorse extracellular flux analyzer in Y_Empty and Y_shGPR81 cells. (I) Basal Respiration, (J) Maximal Respiration and (K) ATP production calculated from OCR data in (H). (L) Representative images of BODIPY FL‐C16 (green) live staining in Y_Empty Y_KD1 and Y_KD2 cells. Nuclei stained using Hoechst 33342 (blue). Scale bar represents 100 μm. (M) Quantification of BODIPY FL‐C16 intensity per cell, normalized to Y_Empty; data shown as for > 100 cells from three independent biological replicates. (N) Immunostaining for CD36 (red) in Y_Empty, Y_KD1 and Y_KD2 myoblasts. Nuclei stained using Hoechst 33342 (blue). Scale bar represents 100 μm. (O) Quantification of CD36 intensity per cell, normalized to Y_Empty; data shown as for > 100 cells from three independent biological replicates. (P) Representative confocal images for BODIPY (green) staining to depict lipid droplets in Y_Empty, Y_KD1 and Y_KD2 myoblasts. Nuclei stained using Hoechst 33342 (blue). Scale bar represents 10 μm. (Q) Quantification of percentage of BODIPY positive cells; data shown for > 100 cells from three independent biological replicates. (R) Immunostaining for PLIN2 (red) in Y_Empty, Y_KD1 and Y_KD2 myoblasts. Nuclei stained using Hoechst 33342 (blue). Scale bar represents 100 μm. (S) Quantification of PLIN2 intensity per cell, normalized to Y_Empty; data shown for > 100 cells from three independent biological replicates. All data shown as mean ± SD. All experiments were performed using three independent biological replicates. *p < 0.05, **p < 0.01, ***p < 0.001.
**Figure S4:** (A) Immunostaining for GPR81 (green) in Y, S, S_CHBA and S_C2 myoblasts. Nuclei stained using Hoechst 33342 (blue). Scale bar represents 100 μm. (B) Quantification of GPR81 intensity per cell, normalized to S; data shown for > 100 cells from three independent biological replicates. (C) Western blot for GPR81 in Y, S, S_CHBA and S_C2 myoblasts, quantified in (D) after normalization to GAPDH (E) Immunostaining for GPR81 (green) in Y, S and S_Lactate myoblasts. Scale bar represents 100 μm. (F) Quantification of GPR81 intensity per cell, normalized to S; data shown for > 100 cells from three independent biological replicates. (G) Representative images of DCFDA (green) live staining in Y, S and S_Lactate cells; scale bar represents 100 μm. (H) Quantification of DCFDA intensity per cell, normalized to S; data shown for > 100 cells from three independent biological replicates. (I) Immunostaining for phosphorylated form of histone H2AX (ϒ‐H2AX) (red) in Y, S and S_Lactate myoblasts; scale bar represents 100 μm; Insets at higher magnification with scale bar = 50 μm. (J) Quantification of percentage of ϒ‐H2AX positive cells. (K) Representative confocal images for Proteostat (red) staining to depict aggresome accumulation; scale bar represents 10 μm. (L) Quantification of fold change in number of proteostat positive cells, normalized to S. (M) Representative images of MitoTracker Red (red) live staining in Y, S, S_Lactate cells. Nuclei stained using Hoechst 33342 (blue). Scale bar represents 100 μm. (N) Quantification of MitoTracker Red intensity per cell, normalized to S; data shown for > 100 cells from three independent biological replicates. Nuclei stained using Hoechst 33342 (blue). (O) Quantitative real‐time PCR to assess the level of mtDNA relative to nDNA (mtDNA/nDNA) using primers specific to the mitochondrial gene *MT‐TL1* and nuclear gene *β2M*. All data shown as mean ± SD. All experiments were performed using three independent biological replicates. *p < 0.05, **p < 0.01, **p < 0.001.
**Figure S5:** (A) Schematic showing experimental timeline; 10 weeks old LAKI mice were administered either Vehicle or CHBA intraperitoneally for 5 weeks, followed by assessment of mouse health and activity. Mice were sacrificed after 5 weeks and muscles were isolated for analysis. (B) Western blot for GPR81 in GA muscle isolated from WT, LAKI and LAKI‐CHBA mice, and (C) quantification of GPR81 expression level after normalization to total protein (Ponceau); WT: *n* = 7, LAKI and LAKI‐CHBA: *n* = 9 biological replicates each. All data shown as mean ± SD. **p < 0.01.
**Figure S6:** (A) Setup of the open field test used to measure activity. Baseline measurements were recorded before the start of injections to record. (B) ambulatory time, (C) ambulatory distance, and (D) ambulatory counts; LAKI: *n* = 13, LAKI‐CHBA: *n* = 12 biological replicates. (E) Western blot for OXPHOS complexes I‐V in GA muscle isolated from WT, LAKI and LAKI‐CHBA mice, and quantification of (F) CI subunit NDUFB8, (G) CII subunit SDHB, (H) CIII subunit UQCRC2, (I) CIV subunit MTCO1, and (J) CV subunit ATP5A expression levels after normalization to total protein (Ponceau); WT: *n* = 7, LAKI and LAKI‐CHBA: *n* = 9 biological replicates each. (K) Citrate synthase activity in GA muscle from LAKI and LAKI‐CHBA mice; *n* = 9 biological replicates each. All data shown as mean ± SD. (L) Representative images of Sudan Black B staining in TA muscles from WT, LAKI and LAKI‐CHBA mice. Scale bar represents 100 μm. ***p < 0.001, ****p < 0.0001.
**Figure S7:** (A) Schematic showing experimental timeline; 10 weeks old LAKI mice were administered either Vehicle or CHBA intraperitoneally for 4 weeks, followed by assessment of muscle force and activity. Thereafter, the right leg of mice was injured using CTX and muscle regeneration was assessed after 1 week, by measuring isometric force produced and muscle histology. (B) Setup of the aurora force transducer used to measure muscle isometric force. Baseline measurements were recorded before the start of injections to record‐ (C) twitch force, (D) maximum tetanic force at a stimulation frequency of 150 Hz, and (E) force‐frequency curve; *n* = 13 biological replicates for each condition. (F) Ratio of twitch force in CTX‐injured to uninjured leg recorded at 1 week after CTX injury. (G) Ratio of maximum tetanic in CTX‐injured to uninjured leg recorded at 1 week after CTX injury; LAKI: *n* = 11, LAKI‐CHBA: *n* = 10 biological replicates. (H) Force frequency curve in Young‐WT mice before and after CTX injury; *n* = 5 biological replicates. All data shown as mean ± SD. *p < 0.05.
**Figure S8:** Uncropped scans of western blots depicted in main figures—(A) Figure 1L, (B) Figure 2D, (C) Figure 6E and (D) Figure 10L.
**Figure S9:** Uncropped scans of western blots depicted in supplemental figures‐ (A) Figure S1A, (B) Figure S1D, (C) Figure S3C, (D) Figure S4B, and (E) Figure S5E.
**Figure S10:** OCR plots normalized only to both cell count and mitochondrial content from (A) Figure 1J‐M, (B) Figure 4J–M, (C) Figure S3H–K, (D) Figure 5A–D, (E) Figure 8C–F, (F) Figure 8G–J. All data shown as mean ± SD for three independent biological replicates. *p < 0.05, **p < 0.01, **p < 0.001, ****p < 0.0001.

## Data Availability

RNA‐seq data generated in this study have been deposited in the NCBI Gene Expression Omnibus (GEO) under accession number GSE325224 and are also available through the NCBI Sequence Read Archive (SRA) under accession number PRJNA1337569. Additional data supporting the findings of this study are available from the corresponding author upon reasonable request.
