# Supplementary material for: Activation of the Lactate Receptor GPR81 Ameliorates Senescence Hallmarks and Improves Muscle Function in Cellular and Progeroid Models of Aging
Source: Aging Cell. 2026 Aug 2;25(8):e70647. doi: 10.1111/acel.70647 (PMC13429102; doi:10.1111/acel.70647)
Supplement: Supplementary file 1 — Figure S1: (A) Flow cytometry histograms for myoblasts stained with BODIPY (green) and detected using Alexa Fluor 488 channel. (B) Quantitative real‐time PCR quantification of mtDNA relative to nDNA (mtDNA/nDNA) as measured by primers specific to mitochondrial gene MT‐TL1 and nuclear gene β2M. (C) Western blot for OxPhos complexes I‐V in Y and S myoblasts. Quantification was performed after normalization to GAPDH for (D) Complex V‐ ATP5A (E) Complex III‐ UQCRC2 (F) Complex II‐ SDHB (G) Complex IV‐ COXII and (H) Complex I‐ NDUFB8. All data shown as mean ± SD. All experiments were performed using three independent biological replicates. **p < 0.01. Figure S2: (A) Western blots for GPR81 protein after knockdown with shGPR81 (Sigma) vector, quantified in (B) after normalization to GAPDH. (C) Quantitative real‐time PCR for GPR81 internally normalized to RPL32 cycle number followed by normalization to Y_Empty. (D) Western blots for GPR81 protein after knockdown with shGPR81 (shLVDP) vector, quantified in (E) after normalization to GAPDH. (F) Senescence‐associated β‐galactosidase (SA‐β‐Gal) staining in Y_Empty, Y_KD1 and Y_KD2 cells. Scale bar represents 100 μm. (G) Quantification for percentage of SA‐β‐Gal cells. (H) Representative images of DCFDA (green) live staining. Scale bar represents 100 μm. (I) Quantification of DCFDA intensity per cell for > 150 cells from three independent biological replicates, normalized to Y_Empty. (J) Immunostaining for phosphorylated form of histone H2AX (ϒ‐H2AX) (red) in Y_Empty, Y_KD1 and Y_KD2 myoblasts. Scale bar represents 100 μm; Insets at higher magnification with scale bar 50 μm. (K) Quantification of percentage of ϒ‐H2AX positive cells. (L) Immunostaining for P21 (red) in Y_Empty, Y_KD1 and Y_KD2 myoblasts; Insets at higher magnification with scale bar 50 μm. (M) Quantification of percentage of P21 positive cells, normalized to Y_Empty. (N) Representative confocal images for Proteostat (red) staining to depict aggresome accumulatio [file ACEL-25-e70647-s001.pptx]

## Slide 1
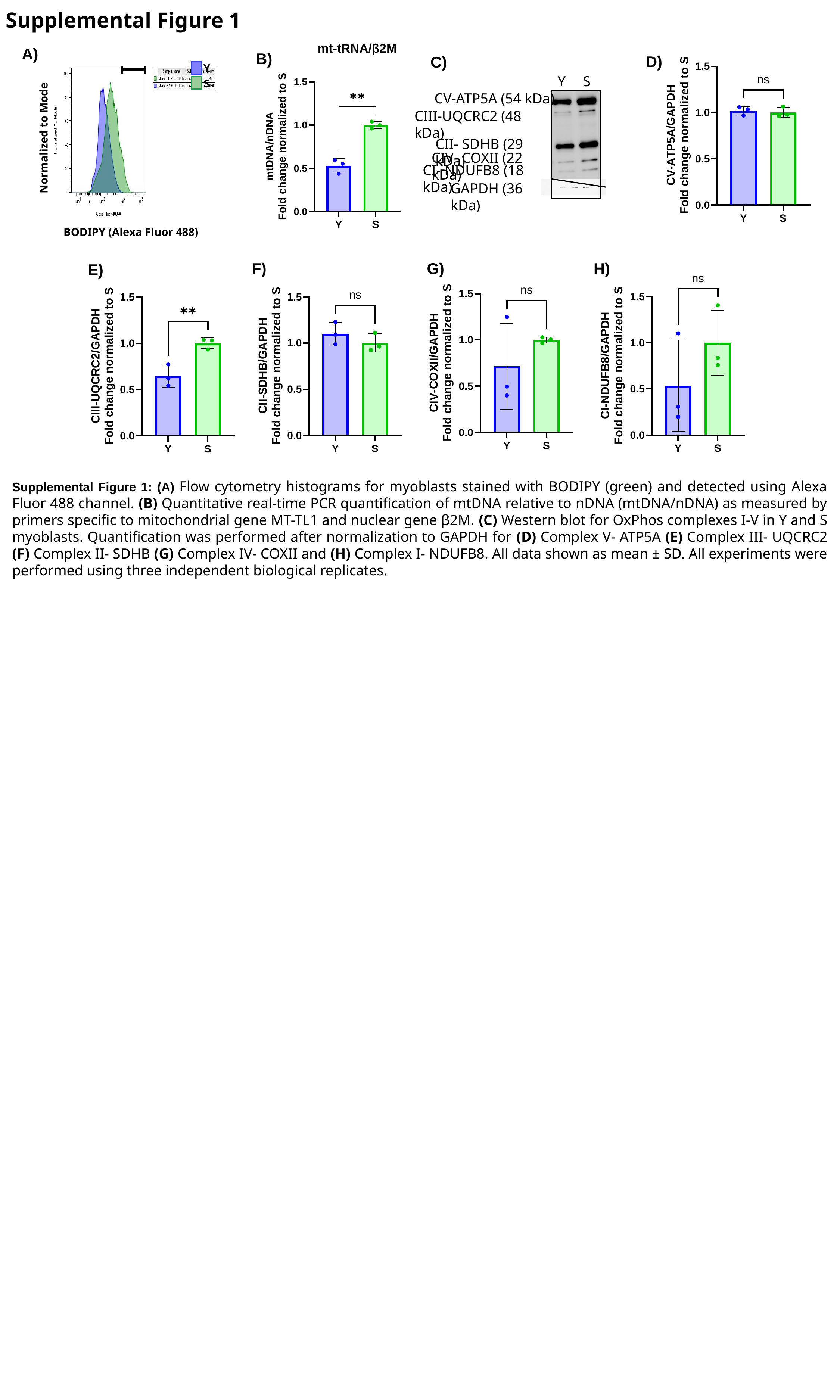

Supplemental Figure 1
A)
B)
D)
C)
Y
S
Normalized to Mode
BODIPY (Alexa Fluor 488)
S
Y
CV-ATP5A (54 kDa)
CIII-UQCRC2 (48 kDa)
CII- SDHB (29 kDa)
CIV- COXII (22 kDa)
CI- NDUFB8 (18 kDa)
GAPDH (36 kDa)
F)
G)
H)
E)
Supplemental Figure 1: (A) Flow cytometry histograms for myoblasts stained with BODIPY (green) and detected using Alexa Fluor 488 channel. (B) Quantitative real-time PCR quantification of mtDNA relative to nDNA (mtDNA/nDNA) as measured by primers specific to mitochondrial gene MT-TL1 and nuclear gene β2M. (C) Western blot for OxPhos complexes I-V in Y and S myoblasts. Quantification was performed after normalization to GAPDH for (D) Complex V- ATP5A (E) Complex III- UQCRC2 (F) Complex II- SDHB (G) Complex IV- COXII and (H) Complex I- NDUFB8. All data shown as mean ± SD. All experiments were performed using three independent biological replicates.

## Slide 2
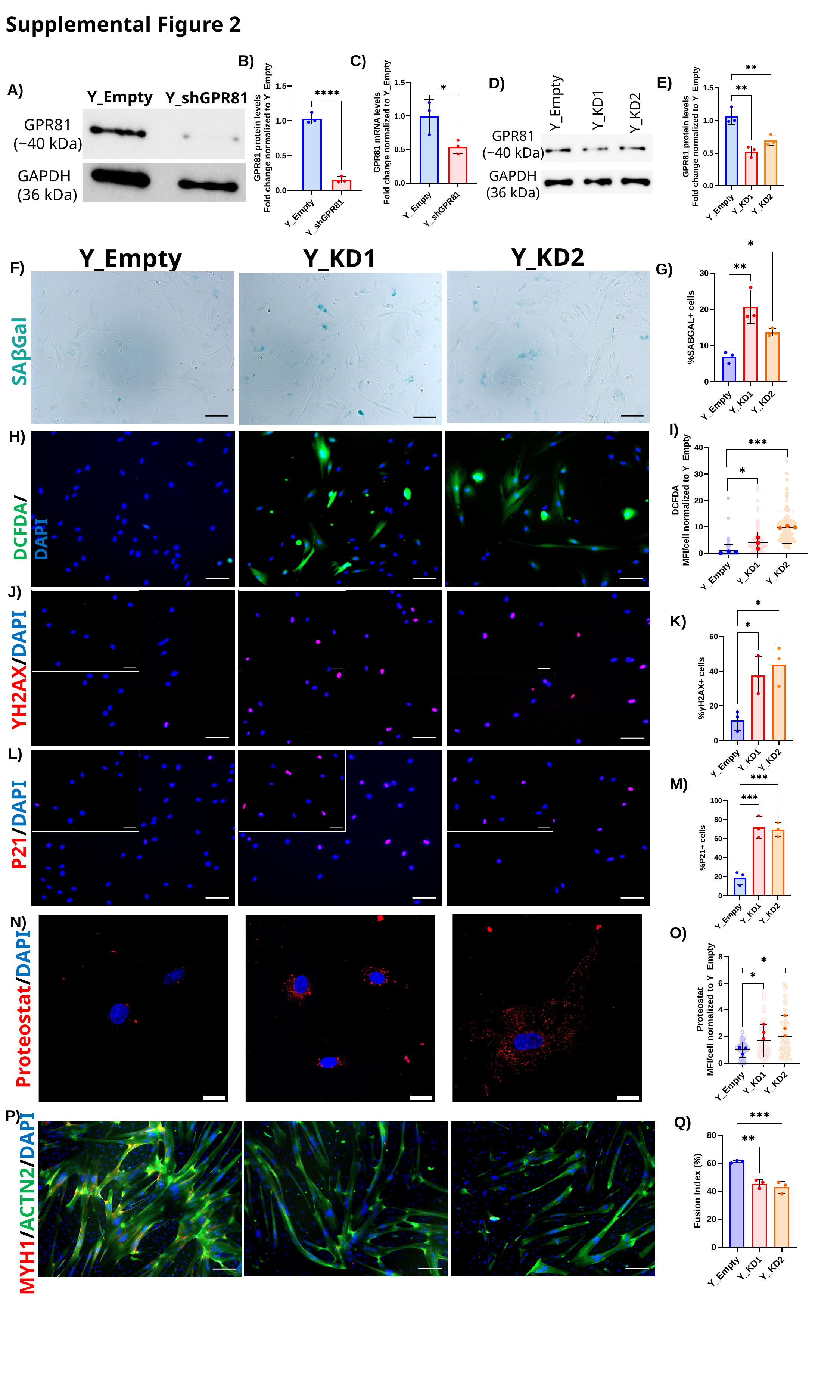

Supplemental Figure 2
B)
C)
E)
D)
A)
Y_Empty
Y_shGPR81
Y_Empty
Y_KD1
Y_KD2
GPR81
(~40 kDa)
GPR81
(~40 kDa)
GAPDH
(36 kDa)
GAPDH
(36 kDa)
Y_KD2
Y_KD1
Y_Empty
F)
G)
SAβGal
I)
H)
DCFDA/DAPI
J)
K)
ϒH2AX/DAPI
L)
M)
P21/DAPI
N)
O)
Proteostat/DAPI
P)
Q)
MYH1/ACTN2/DAPI

## Slide 3
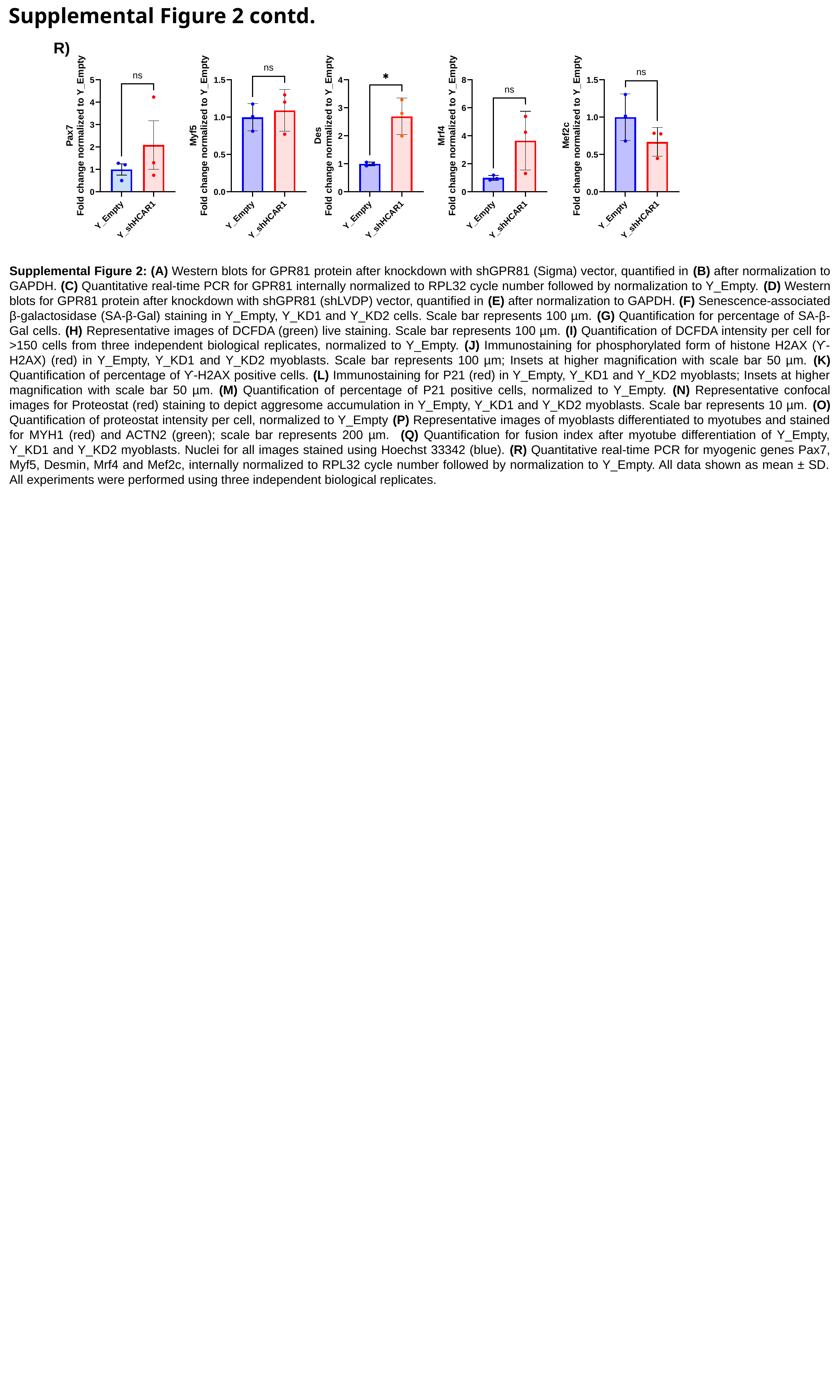

Supplemental Figure 2 contd.
R)
Supplemental Figure 2: (A) Western blots for GPR81 protein after knockdown with shGPR81 (Sigma) vector, quantified in (B) after normalization to GAPDH. (C) Quantitative real-time PCR for GPR81 internally normalized to RPL32 cycle number followed by normalization to Y_Empty. (D) Western blots for GPR81 protein after knockdown with shGPR81 (shLVDP) vector, quantified in (E) after normalization to GAPDH. (F) Senescence-associated β-galactosidase (SA-β-Gal) staining in Y_Empty, Y_KD1 and Y_KD2 cells. Scale bar represents 100 µm. (G) Quantification for percentage of SA-β-Gal cells. (H) Representative images of DCFDA (green) live staining. Scale bar represents 100 µm. (I) Quantification of DCFDA intensity per cell for >150 cells from three independent biological replicates, normalized to Y_Empty. (J) Immunostaining for phosphorylated form of histone H2AX (ϒ-H2AX) (red) in Y_Empty, Y_KD1 and Y_KD2 myoblasts. Scale bar represents 100 µm; Insets at higher magnification with scale bar 50 µm. (K) Quantification of percentage of ϒ-H2AX positive cells. (L) Immunostaining for P21 (red) in Y_Empty, Y_KD1 and Y_KD2 myoblasts; Insets at higher magnification with scale bar 50 µm. (M) Quantification of percentage of P21 positive cells, normalized to Y_Empty. (N) Representative confocal images for Proteostat (red) staining to depict aggresome accumulation in Y_Empty, Y_KD1 and Y_KD2 myoblasts. Scale bar represents 10 µm. (O) Quantification of proteostat intensity per cell, normalized to Y_Empty (P) Representative images of myoblasts differentiated to myotubes and stained for MYH1 (red) and ACTN2 (green); scale bar represents 200 µm. (Q) Quantification for fusion index after myotube differentiation of Y_Empty, Y_KD1 and Y_KD2 myoblasts. Nuclei for all images stained using Hoechst 33342 (blue). (R) Quantitative real-time PCR for myogenic genes Pax7, Myf5, Desmin, Mrf4 and Mef2c, internally normalized to RPL32 cycle number followed by normalization to Y_Empty. All data shown as mean ± SD. All experiments were performed using three independent biological replicates.

## Slide 4
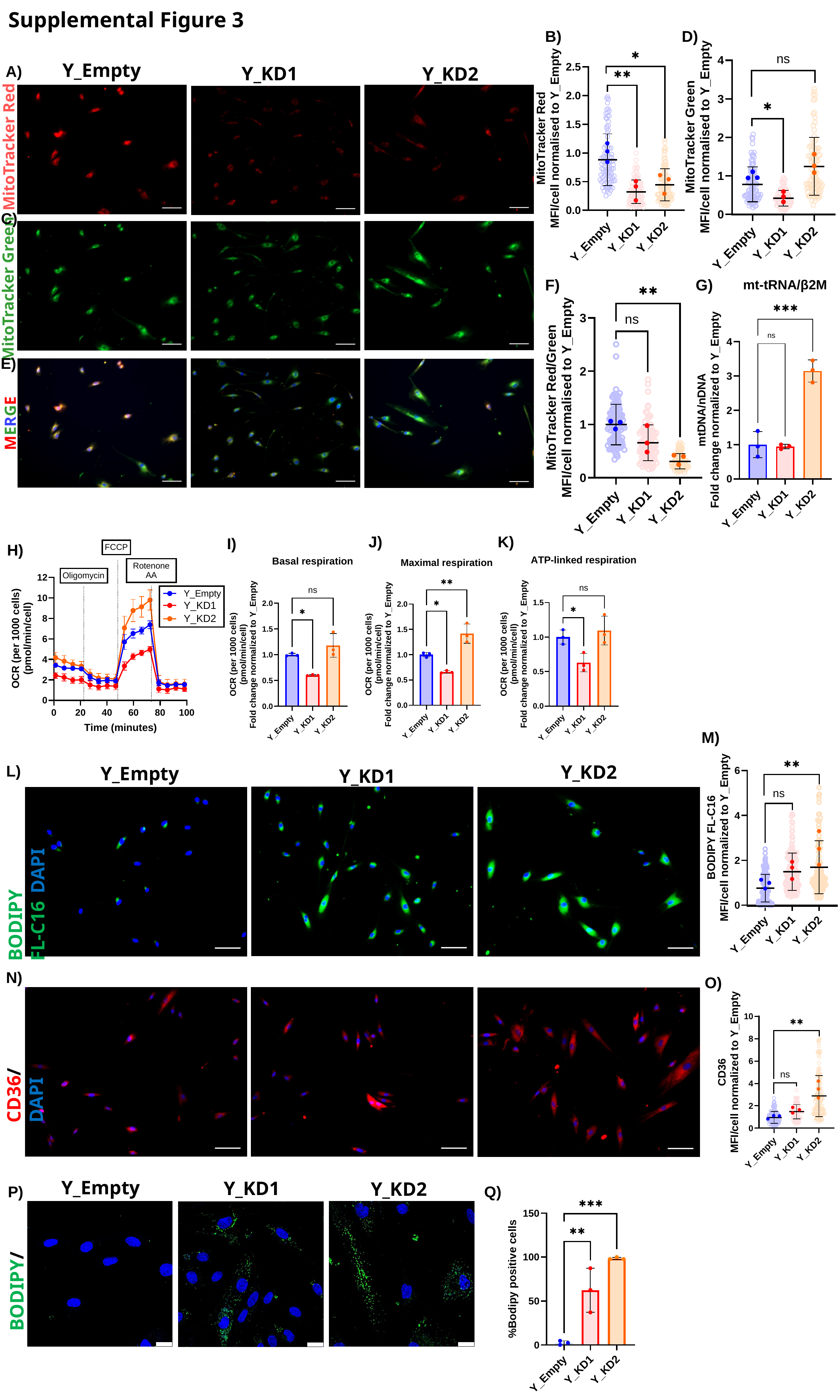

Supplemental Figure 3
B)
D)
Y_Empty
A)
Y_KD1
Y_KD2
MitoTracker Red
C)
F)
G)
MitoTracker Green
E)
MERGE
J)
K)
I)
H)
M)
Y_KD2
L)
Y_Empty
Y_KD1
BODIPY FL-C16/DAPI
N)
O)
CD36/DAPI
Y_Empty
Y_KD1
Y_KD2
P)
Q)
BODIPY/DAPI

## Slide 5
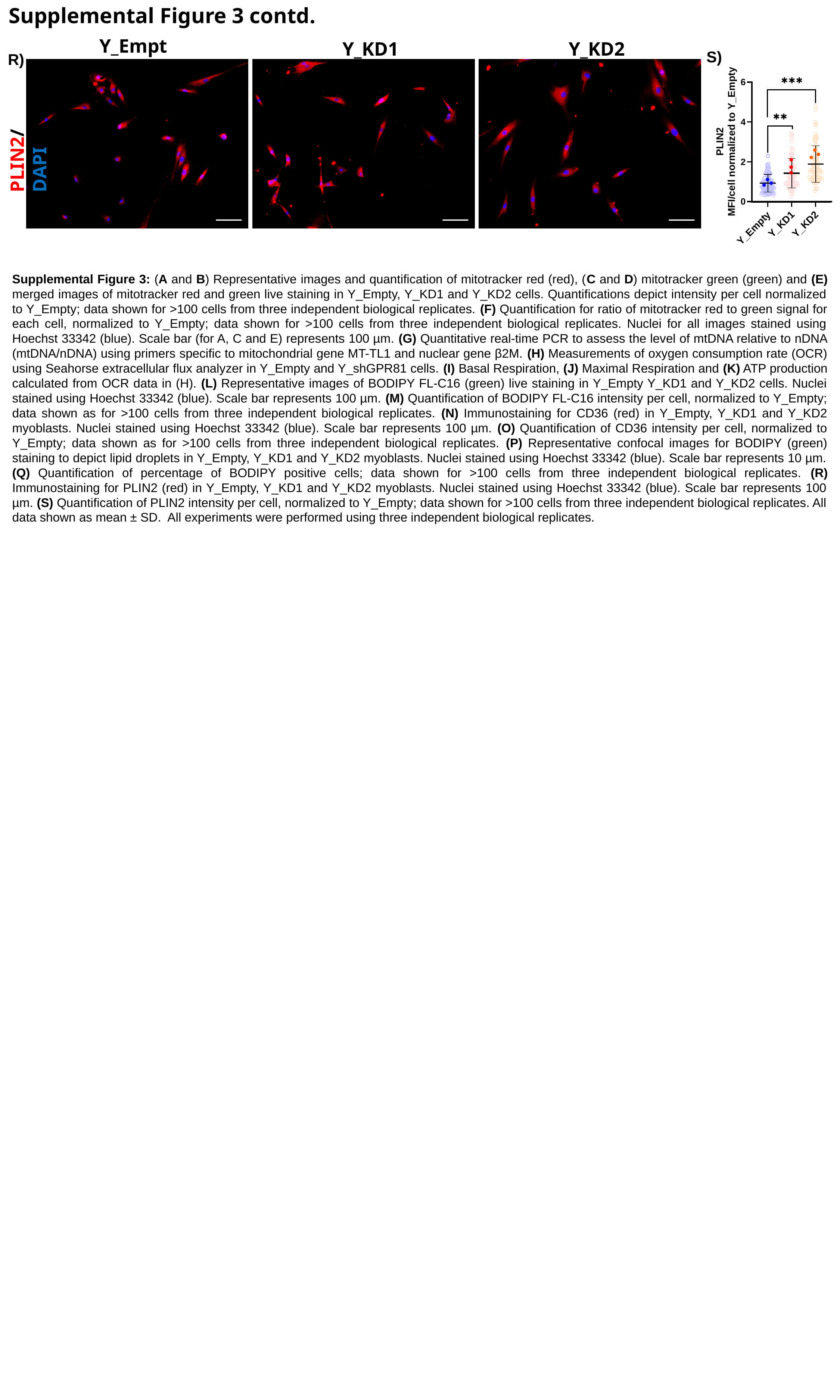

Supplemental Figure 3 contd.
Y_Empty
Y_KD1
Y_KD2
S)
R)
PLIN2/DAPI
Supplemental Figure 3: (A and B) Representative images and quantification of mitotracker red (red), (C and D) mitotracker green (green) and (E) merged images of mitotracker red and green live staining in Y_Empty, Y_KD1 and Y_KD2 cells. Quantifications depict intensity per cell normalized to Y_Empty; data shown for >100 cells from three independent biological replicates. (F) Quantification for ratio of mitotracker red to green signal for each cell, normalized to Y_Empty; data shown for >100 cells from three independent biological replicates. Nuclei for all images stained using Hoechst 33342 (blue). Scale bar (for A, C and E) represents 100 µm. (G) Quantitative real-time PCR to assess the level of mtDNA relative to nDNA (mtDNA/nDNA) using primers specific to mitochondrial gene MT-TL1 and nuclear gene β2M. (H) Measurements of oxygen consumption rate (OCR) using Seahorse extracellular flux analyzer in Y_Empty and Y_shGPR81 cells. (I) Basal Respiration, (J) Maximal Respiration and (K) ATP production calculated from OCR data in (H). (L) Representative images of BODIPY FL-C16 (green) live staining in Y_Empty Y_KD1 and Y_KD2 cells. Nuclei stained using Hoechst 33342 (blue). Scale bar represents 100 µm. (M) Quantification of BODIPY FL-C16 intensity per cell, normalized to Y_Empty; data shown as for >100 cells from three independent biological replicates. (N) Immunostaining for CD36 (red) in Y_Empty, Y_KD1 and Y_KD2 myoblasts. Nuclei stained using Hoechst 33342 (blue). Scale bar represents 100 µm. (O) Quantification of CD36 intensity per cell, normalized to Y_Empty; data shown as for >100 cells from three independent biological replicates. (P) Representative confocal images for BODIPY (green) staining to depict lipid droplets in Y_Empty, Y_KD1 and Y_KD2 myoblasts. Nuclei stained using Hoechst 33342 (blue). Scale bar represents 10 µm. (Q) Quantification of percentage of BODIPY positive cells; data shown for >100 cells from three independent biological replicates. (R) Immunostaining for PLIN2 (red) in Y_Empty, Y_KD1 and Y_KD2 myoblasts. Nuclei stained using Hoechst 33342 (blue). Scale bar represents 100 µm. (S) Quantification of PLIN2 intensity per cell, normalized to Y_Empty; data shown for >100 cells from three independent biological replicates. All data shown as mean ± SD. All experiments were performed using three independent biological replicates.

## Slide 6
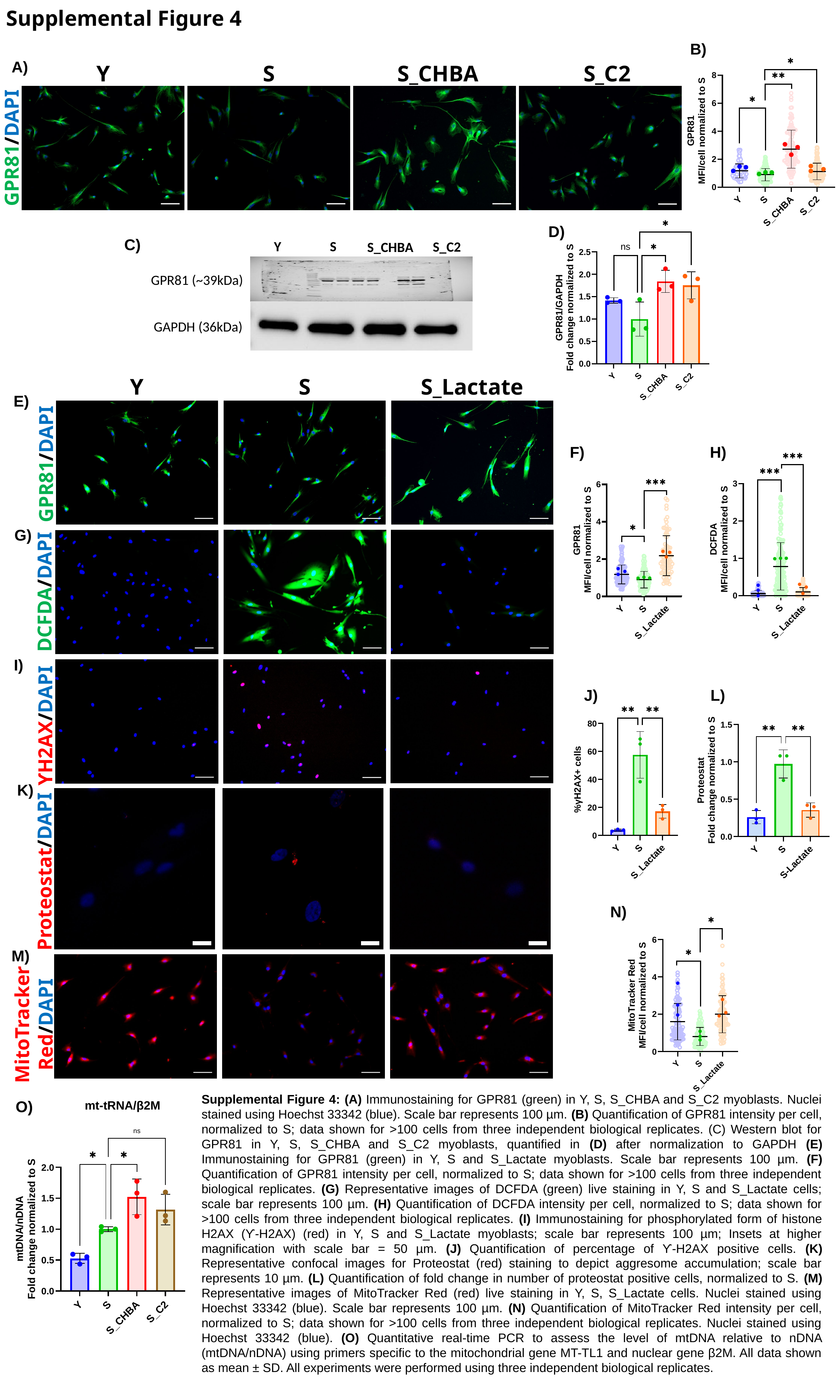

Supplemental Figure 4
B)
A)
Y
S
S_C2
S_CHBA
GPR81/DAPI
D)
C)
Y
S
S_C2
S_CHBA
GPR81 (~39kDa)
GAPDH (36kDa)
Y
S
S_Lactate
E)
F)
H)
GPR81/DAPI
G)
DCFDA/DAPI
I)
J)
L)
ϒH2AX/DAPI
K)
Proteostat/DAPI
N)
M)
MitoTracker Red/DAPI
Supplemental Figure 4: (A) Immunostaining for GPR81 (green) in Y, S, S_CHBA and S_C2 myoblasts. Nuclei stained using Hoechst 33342 (blue). Scale bar represents 100 µm. (B) Quantification of GPR81 intensity per cell, normalized to S; data shown for >100 cells from three independent biological replicates. (C) Western blot for GPR81 in Y, S, S_CHBA and S_C2 myoblasts, quantified in (D) after normalization to GAPDH (E) Immunostaining for GPR81 (green) in Y, S and S_Lactate myoblasts. Scale bar represents 100 µm. (F) Quantification of GPR81 intensity per cell, normalized to S; data shown for >100 cells from three independent biological replicates. (G) Representative images of DCFDA (green) live staining in Y, S and S_Lactate cells; scale bar represents 100 µm. (H) Quantification of DCFDA intensity per cell, normalized to S; data shown for >100 cells from three independent biological replicates. (I) Immunostaining for phosphorylated form of histone H2AX (ϒ-H2AX) (red) in Y, S and S_Lactate myoblasts; scale bar represents 100 µm; Insets at higher magnification with scale bar = 50 µm. (J) Quantification of percentage of ϒ-H2AX positive cells. (K) Representative confocal images for Proteostat (red) staining to depict aggresome accumulation; scale bar represents 10 µm. (L) Quantification of fold change in number of proteostat positive cells, normalized to S. (M) Representative images of MitoTracker Red (red) live staining in Y, S, S_Lactate cells. Nuclei stained using Hoechst 33342 (blue). Scale bar represents 100 µm. (N) Quantification of MitoTracker Red intensity per cell, normalized to S; data shown for >100 cells from three independent biological replicates. Nuclei stained using Hoechst 33342 (blue). (O) Quantitative real-time PCR to assess the level of mtDNA relative to nDNA (mtDNA/nDNA) using primers specific to the mitochondrial gene MT-TL1 and nuclear gene β2M. All data shown as mean ± SD. All experiments were performed using three independent biological replicates.
O)

## Slide 7
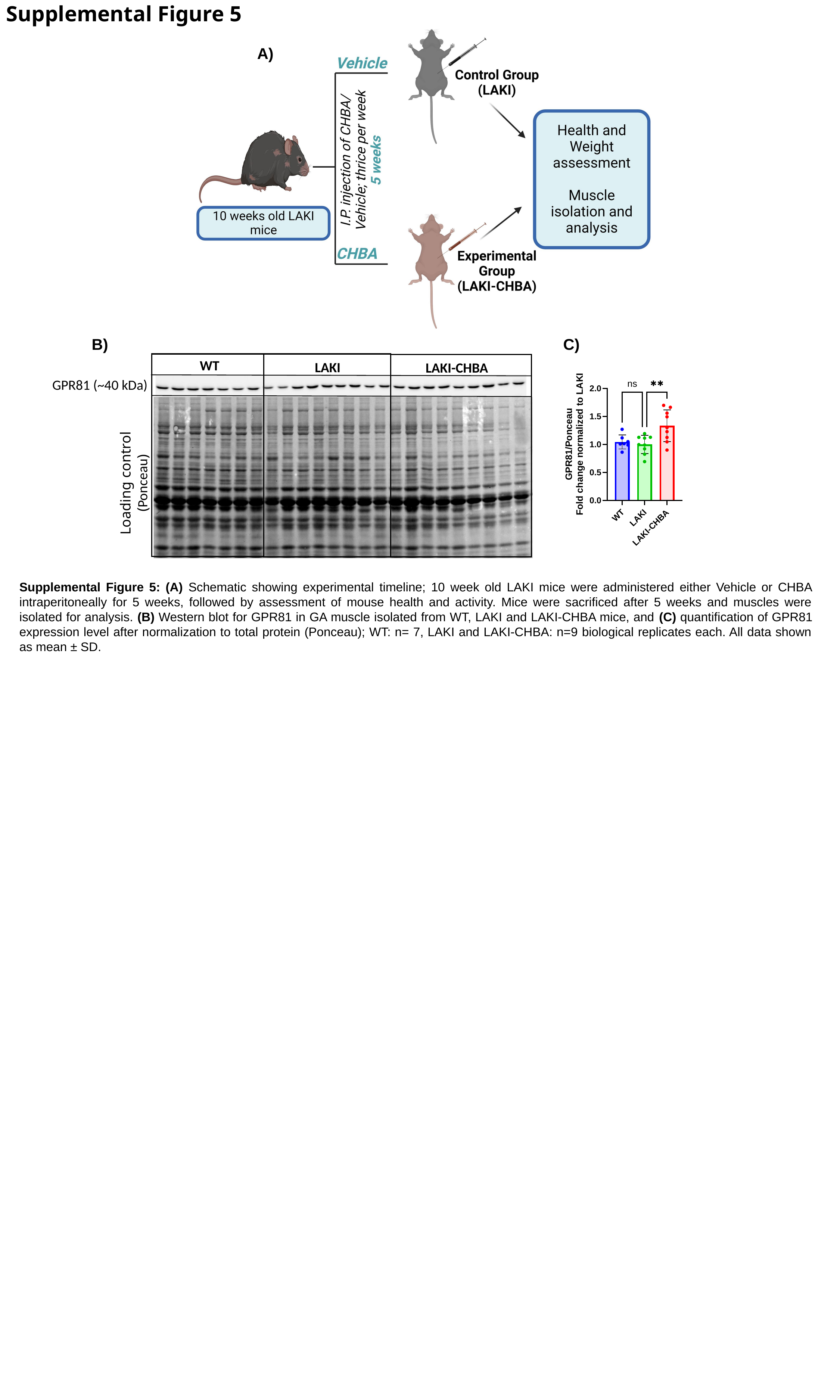

Supplemental Figure 5
A)
B)
C)
WT
LAKI
LAKI-CHBA
GPR81 (~40 kDa)
Loading control (Ponceau)
Supplemental Figure 5: (A) Schematic showing experimental timeline; 10 week old LAKI mice were administered either Vehicle or CHBA intraperitoneally for 5 weeks, followed by assessment of mouse health and activity. Mice were sacrificed after 5 weeks and muscles were isolated for analysis. (B) Western blot for GPR81 in GA muscle isolated from WT, LAKI and LAKI-CHBA mice, and (C) quantification of GPR81 expression level after normalization to total protein (Ponceau); WT: n= 7, LAKI and LAKI-CHBA: n=9 biological replicates each. All data shown as mean ± SD.

## Slide 8
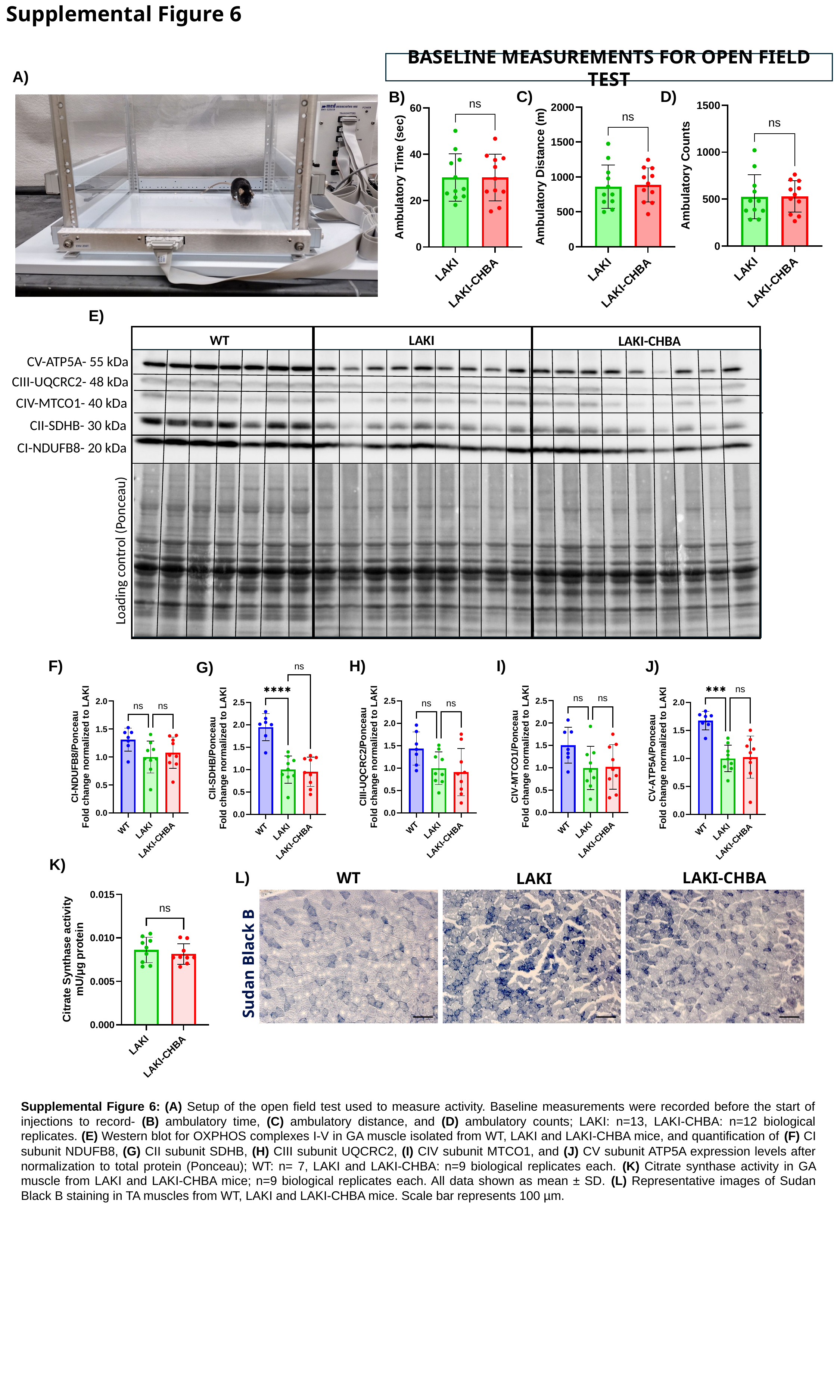

Supplemental Figure 6
BASELINE MEASUREMENTS FOR OPEN FIELD TEST
A)
C)
D)
B)
E)
WT
LAKI
LAKI-CHBA
CV-ATP5A- 55 kDa
CIII-UQCRC2- 48 kDa
CIV-MTCO1- 40 kDa
CII-SDHB- 30 kDa
CI-NDUFB8- 20 kDa
Loading control (Ponceau)
F)
H)
I)
J)
G)
K)
L)
WT
LAKI-CHBA
LAKI
Sudan Black B
Supplemental Figure 6: (A) Setup of the open field test used to measure activity. Baseline measurements were recorded before the start of injections to record- (B) ambulatory time, (C) ambulatory distance, and (D) ambulatory counts; LAKI: n=13, LAKI-CHBA: n=12 biological replicates. (E) Western blot for OXPHOS complexes I-V in GA muscle isolated from WT, LAKI and LAKI-CHBA mice, and quantification of (F) CI subunit NDUFB8, (G) CII subunit SDHB, (H) CIII subunit UQCRC2, (I) CIV subunit MTCO1, and (J) CV subunit ATP5A expression levels after normalization to total protein (Ponceau); WT: n= 7, LAKI and LAKI-CHBA: n=9 biological replicates each. (K) Citrate synthase activity in GA muscle from LAKI and LAKI-CHBA mice; n=9 biological replicates each. All data shown as mean ± SD. (L) Representative images of Sudan Black B staining in TA muscles from WT, LAKI and LAKI-CHBA mice. Scale bar represents 100 µm.

## Slide 9
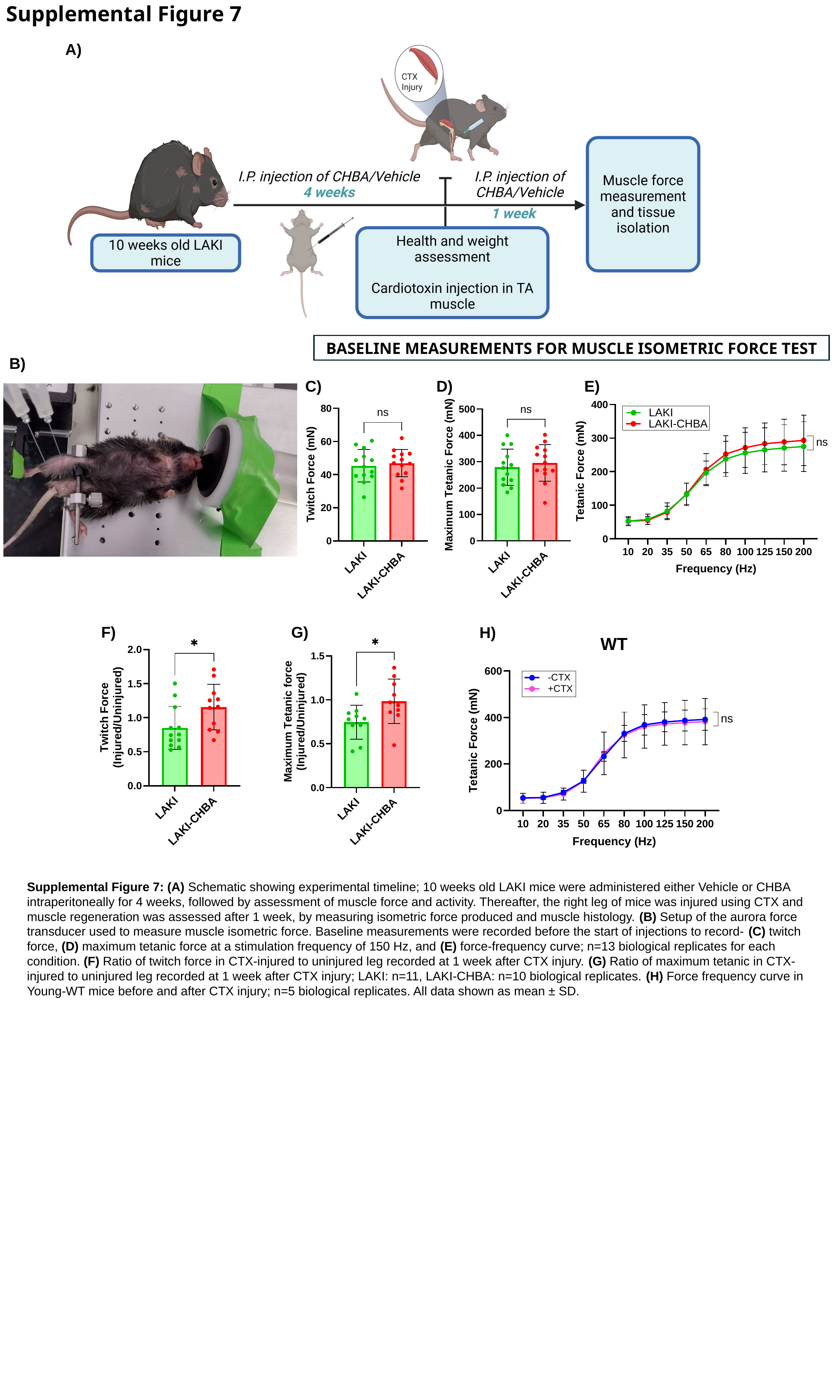

Supplemental Figure 7
A)
BASELINE MEASUREMENTS FOR MUSCLE ISOMETRIC FORCE TEST
B)
C)
D)
E)
G)
F)
H)
Supplemental Figure 7: (A) Schematic showing experimental timeline; 10 weeks old LAKI mice were administered either Vehicle or CHBA intraperitoneally for 4 weeks, followed by assessment of muscle force and activity. Thereafter, the right leg of mice was injured using CTX and muscle regeneration was assessed after 1 week, by measuring isometric force produced and muscle histology. (B) Setup of the aurora force transducer used to measure muscle isometric force. Baseline measurements were recorded before the start of injections to record- (C) twitch force, (D) maximum tetanic force at a stimulation frequency of 150 Hz, and (E) force-frequency curve; n=13 biological replicates for each condition. (F) Ratio of twitch force in CTX-injured to uninjured leg recorded at 1 week after CTX injury. (G) Ratio of maximum tetanic in CTX-injured to uninjured leg recorded at 1 week after CTX injury; LAKI: n=11, LAKI-CHBA: n=10 biological replicates. (H) Force frequency curve in Young-WT mice before and after CTX injury; n=5 biological replicates. All data shown as mean ± SD.

## Slide 10
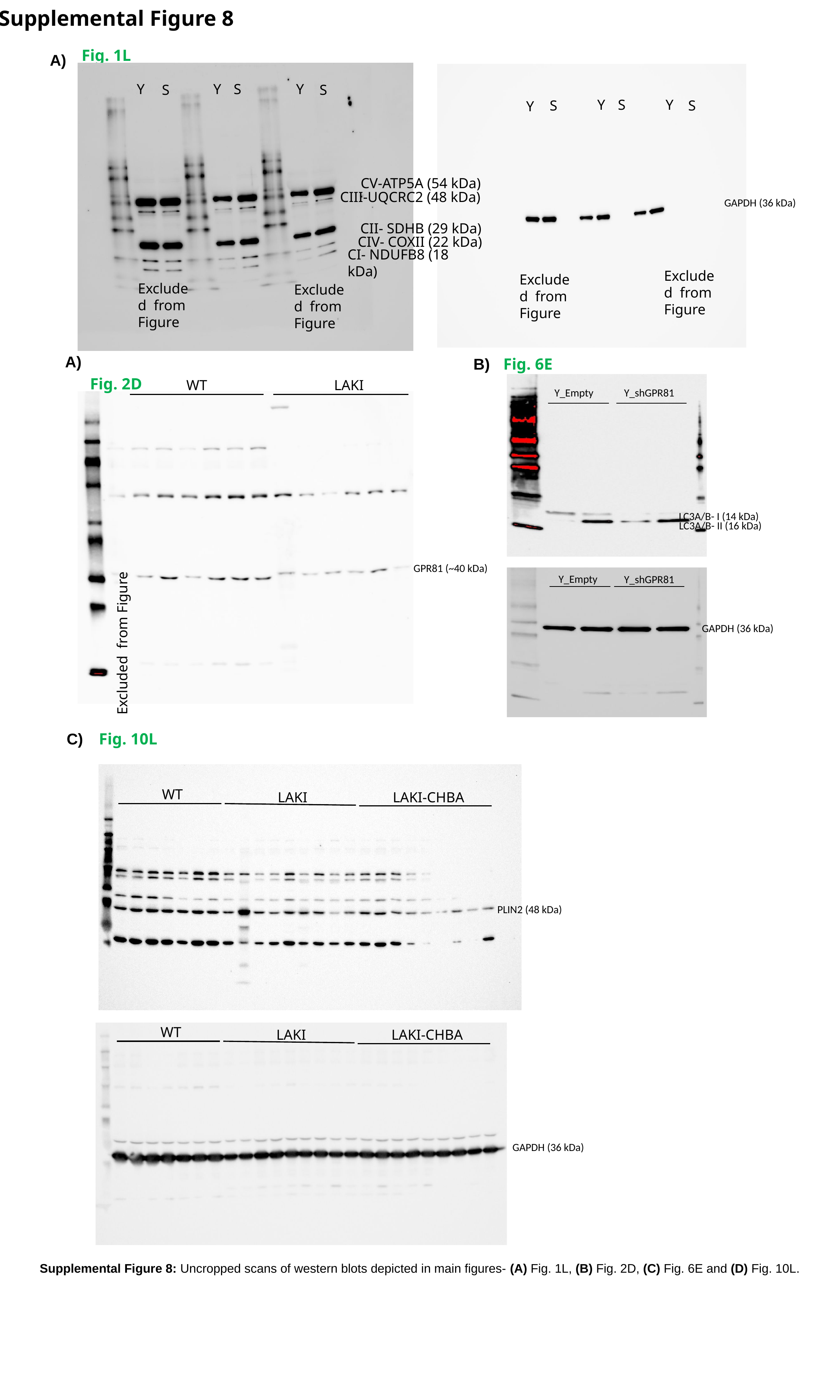

Supplemental Figure 8
Fig. 1L
A)
Y
Y
S
Y
S
S
Y
S
Y
S
S
Y
CV-ATP5A (54 kDa)
CIII-UQCRC2 (48 kDa)
GAPDH (36 kDa)
CII- SDHB (29 kDa)
CIV- COXII (22 kDa)
CI- NDUFB8 (18 kDa)
Excluded from Figure
Excluded from Figure
Excluded from Figure
Excluded from Figure
A)
B)
Fig. 6E
Fig. 2D
WT
LAKI
Y_Empty
Y_shGPR81
LC3A/B- I (14 kDa)
LC3A/B- II (16 kDa)
GPR81 (~40 kDa)
Excluded from Figure
Y_Empty
Y_shGPR81
GAPDH (36 kDa)
C)
Fig. 10L
WT
LAKI
LAKI-CHBA
PLIN2 (48 kDa)
WT
LAKI
LAKI-CHBA
GAPDH (36 kDa)
Supplemental Figure 8: Uncropped scans of western blots depicted in main figures- (A) Fig. 1L, (B) Fig. 2D, (C) Fig. 6E and (D) Fig. 10L.

## Slide 11
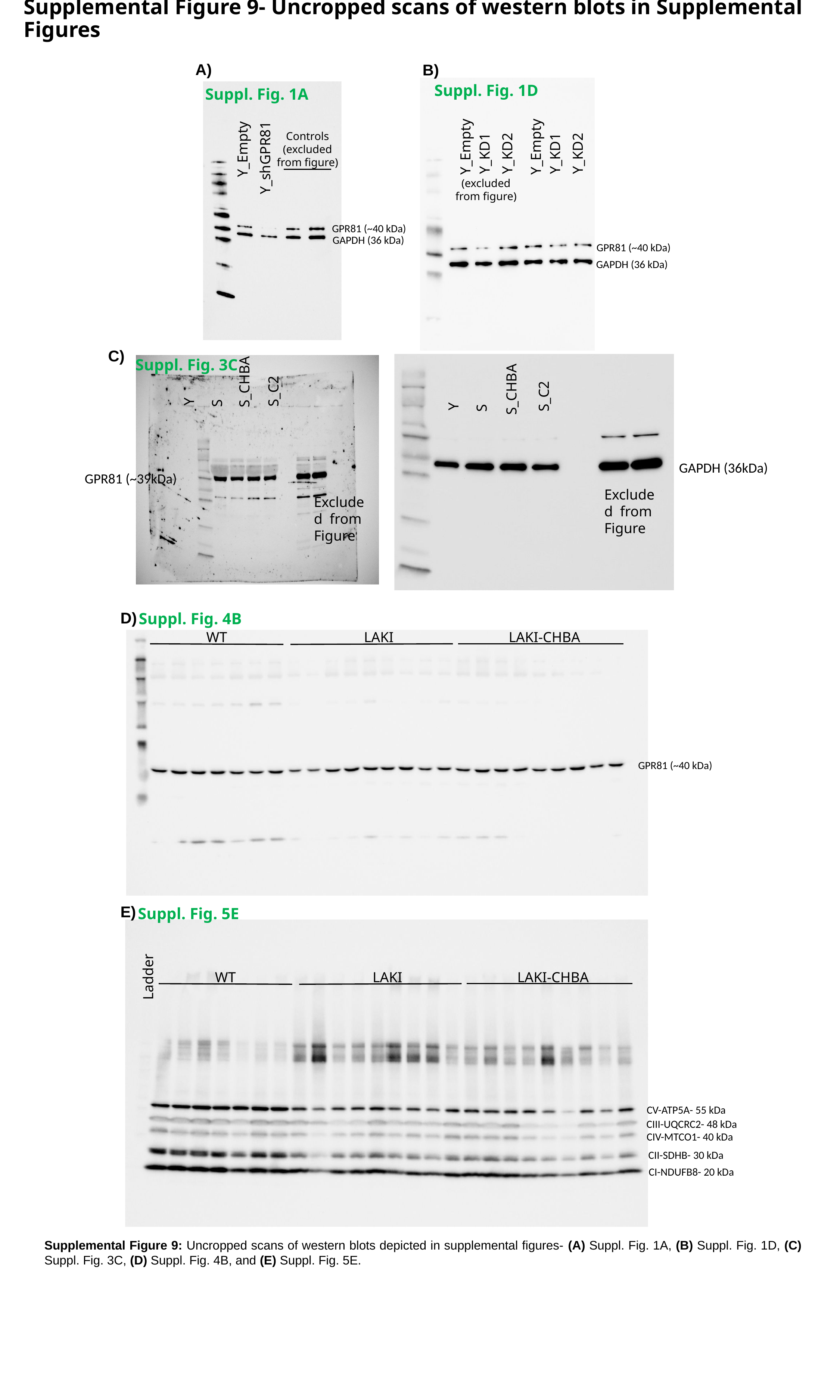

Supplemental Figure 9- Uncropped scans of western blots in Supplemental Figures
A)
B)
Suppl. Fig. 1D
Suppl. Fig. 1A
Controls (excluded from figure)
Y_Empty
Y_Empty
Y_Empty
Y_KD1
Y_KD2
Y_KD1
Y_KD2
Y_shGPR81
(excluded from figure)
GPR81 (~40 kDa)
GAPDH (36 kDa)
GPR81 (~40 kDa)
GAPDH (36 kDa)
C)
Suppl. Fig. 3C
S_CHBA
S_CHBA
S_C2
S_C2
Y
S
Y
S
GAPDH (36kDa)
GPR81 (~39kDa)
Excluded from Figure
Excluded from Figure
D)
Suppl. Fig. 4B
WT
LAKI
LAKI-CHBA
GPR81 (~40 kDa)
E)
Suppl. Fig. 5E
Ladder
WT
LAKI
LAKI-CHBA
CV-ATP5A- 55 kDa
CIII-UQCRC2- 48 kDa
CIV-MTCO1- 40 kDa
CII-SDHB- 30 kDa
CI-NDUFB8- 20 kDa
Supplemental Figure 9: Uncropped scans of western blots depicted in supplemental figures- (A) Suppl. Fig. 1A, (B) Suppl. Fig. 1D, (C) Suppl. Fig. 3C, (D) Suppl. Fig. 4B, and (E) Suppl. Fig. 5E.

## Slide 12
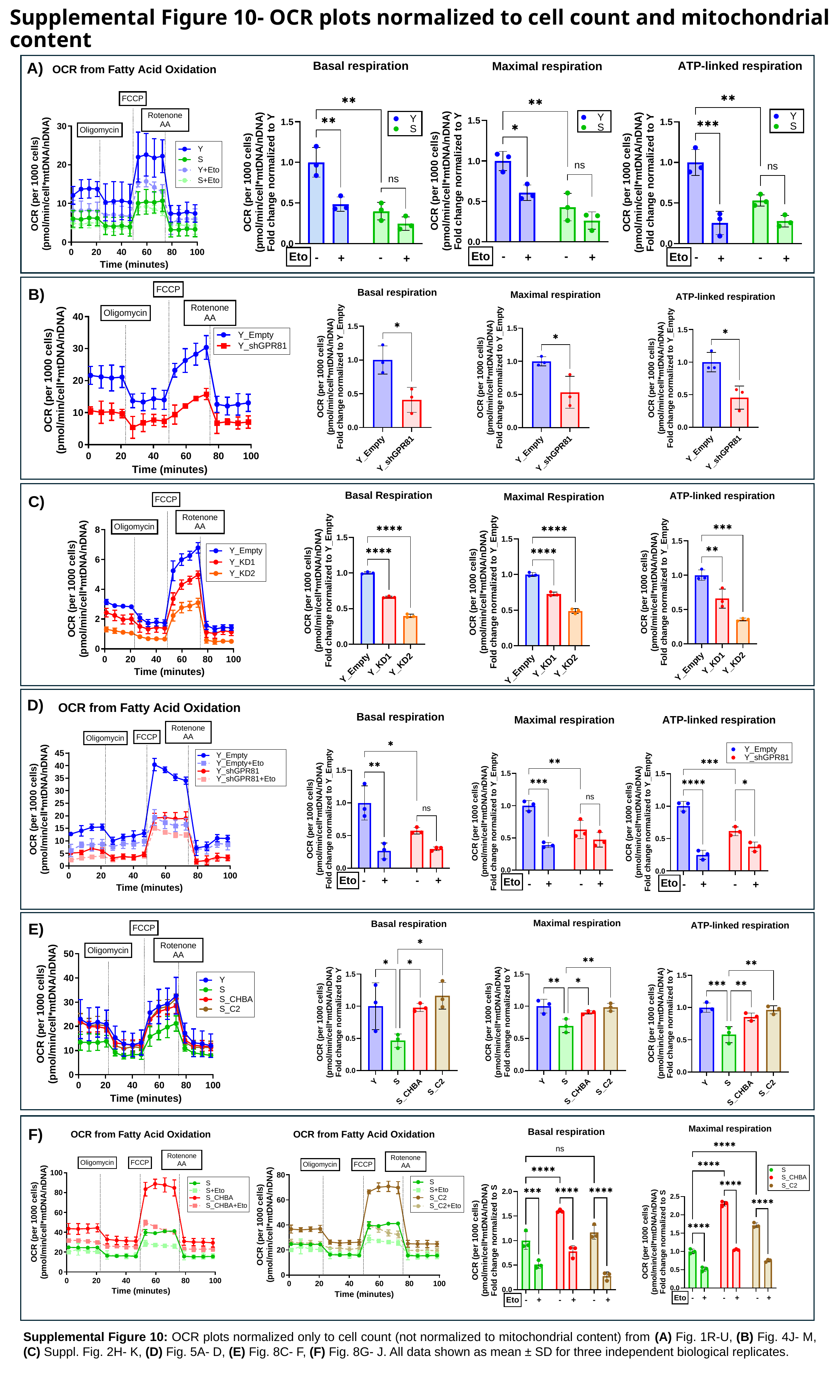

Supplemental Figure 10- OCR plots normalized to cell count and mitochondrial content
A)
B)
C)
D)
E)
F)
Supplemental Figure 10: OCR plots normalized only to cell count (not normalized to mitochondrial content) from (A) Fig. 1R-U, (B) Fig. 4J- M, (C) Suppl. Fig. 2H- K, (D) Fig. 5A- D, (E) Fig. 8C- F, (F) Fig. 8G- J. All data shown as mean ± SD for three independent biological replicates.
